# The impact of post-acute sequelae of COVID-19 on cardiac function and structure: A systematic review and a hybrid individual participant data meta-analysis

**DOI:** 10.1016/j.ajpc.2026.101457

**Published:** 2026-01-31

**Authors:** Binyam Tariku Seboka, Lillian Ma, Dianna J. Magliano, Stella Talic, Antonio da Silva Menezes Junior, Alessandra Borlotti, Helena Thomaides Brears, Andrea Dennis, Amitava Banerjee, Thomas H. Marwick, Quan Huynh

**Affiliations:** a Baker Department of Cardiometabolic Health, University of Melbourne, Melbourne, Victoria, Australia; b Imaging Research Laboratory, Baker Heart and Diabetes Institute, Melbourne, Victoria, Australia; c Diabetes and Population Health, Baker Heart and Diabetes Institute, Melbourne, Australia; d School of Public Health and Preventive Medicine, Monash University, Melbourne, Australia; e Monash University, Clinical Epidemiology and Pharmacoepidemiology, Australia; f Faculty of Medicine, Federal University of Goiás, Brazil; g Perspectum Diagnostics, United Kingdom; h Institute of Health Informatics, University College London, United Kingdom; i Menzies Institute for Medical Research, University of Tasmania, Hobart, Australia

**Keywords:** Post-acute sequelae of SARS-CoV-2 infection (PASC), Cardiac function, Cardiac structure, Long COVID-19, Systematic review

## Abstract

**Background:**

Post-acute sequelae of SARS-CoV-2 infection (PASC), also referred to as Long COVID, affect a significant proportion of COVID-19 survivors, with evidence suggesting cardiovascular involvement. However, the nature, extent, and clinical significance of these alterations remain uncertain.

**Aim:**

To synthesize evidence on structural and functional cardiac alterations in individuals with PASC compared with those without PASC.

**Method:**

We systematically searched seven databases. Random-effects meta-analyses were performed and supplemented by individual participant data (IPD) analyses from three studies. Univariable and multivariable meta-regressions examined associations with study-level characteristics. Publication bias and evidence certainty were assessed using standard methods (funnel plots, Egger’s test, trim-and-fill, and GRADE).

**Results:**

From 3580 records, 17 studies with 4852 participants (3173 PASC, 1679 controls) met the inclusion criteria. IPD analysis revealed an impairment in global longitudinal strain (GLS) (mean difference (MD) = 3.63 %) in individuals with PASC. When using categorical thresholds, 58 % of individuals with PASC had GLS < 16 %, indicating a significant prevalence of subclinical left ventricular dysfunction. Meta-analysis supported these findings, showing impaired GLS (MD = 1.07 %), along with reductions in left ventricular ejection fraction (MD = -1.30 %) and left ventricular end-diastolic volume (MD=-3.98 mL). Meta-regression showed that cardiac dysfunction was more frequently observed in individuals with older age, diabetes, and hypertension.

**Conclusion:**

This review indicates that PASC is associated with modest, subclinical alterations in cardiac function. These alterations appear more pronounced in older adults and those with cardiometabolic comorbidities, highlighting the potential value of risk-stratified cardiovascular surveillance in individuals with PASC. The long-term clinical relevance of these changes remains unclear and warrants further study.

## Highlights

• This study examines the impact of persistent symptoms following COVID-19 infection (known as Post-Acute Sequelae of SARS-CoV-2 infection or PASC) on heart function.
• Individuals with PASC show measurable impairments in cardiac function, particularly in global longitudinal strain (GLS) and left ventricular ejection fraction (LVEF).
• Cardiac abnormalities are more pronounced in older adults and in those with diabetes or hypertension, indicating increased cardiovascular vulnerability and supporting a risk-stratified surveillance approach.

## Central illustration

**Figure:**
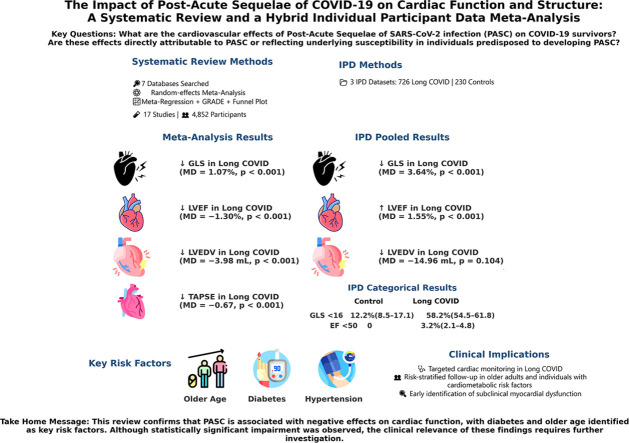

## 1 Introduction

Severe Acute Respiratory Syndrome Coronavirus 2 (SARS-CoV-2) infection has been shown to have effects that extend beyond the acute phase of illness. By December 2025, >777 million people worldwide had been infected with COVID-19, and 10–20 % were estimated to develop post-acute sequelae of SARS-CoV-2 (PASC) [[1], [2], [3]]. This burden is expected to increase as the virus continues to infect tens of thousands of people globally each week [1]. Despite the respiratory onset of COVID-19, myocardial injury, arrhythmias, and ventricular dysfunction were commonly documented during both the acute phase and recovery [[4], [5], [6], [7]], raising concerns that PASC may also lead to long-term cardiac sequelae.

PASC, also referred to as Long COVID, is defined by the World Health Organization (WHO) as the persistence or emergence of symptoms at least three months after infection, lasting for a minimum of two months without an alternative explanation [8]. While cardiovascular symptoms such as palpitations, arrhythmias, chest pain, and dysautonomia are commonly reported in PASC [[9], [10], [11],^12^], understanding its impact on cardiac function and structure require objective assessment. This may requires assessment through imaging modalities such as echocardiography, cardiac magnetic resonance imaging (CMR), and other advanced techniques [13,14]. Emerging evidence points to alterations in myocardial performance, ventricular remodeling, and subclinical cardiac injury [[15], [16], [17]]. The potential of these effects could resemble the multiple-hit hypothesis, where an initial myocardial injury may predispose individuals to longer-term cardiac dysfunction [18,19]. Whether these changes are directly attributable to PASC or reflect underlying susceptibility in individuals predisposed to developing PASC remains uncertain, and there is limited consensus on the persistence and progression of these alterations over time.

Existing systematic reviews and meta-analyses characterized the cardiac phenotype during the acute and after recovery phases of COVID-19, including myocardial injury, left and right ventricular dysfunction [[20], [21], [22]]. However, PASC represents a distinct post-infectious condition, and it is unclear how the cardiovascular sequelae evolve in this population. Hence, this systematic review and hybrid individual participant data (IPD) meta-analysis aimed to synthesize existing evidence on cardiac structural and functional changes in individuals with PASC. By combining aggregate and participant-level data, we sought to provide a comprehensive understanding of its long-term impact on cardiovascular health and to inform risk-stratified monitoring and future research priorities in this growing population.

## 2 Methods

**Study design.** The study protocol was prospectively registered in the PROSPERO (CRD42024577867) [23]. The review followed the Meta-analysis of Observational Studies in Epidemiology (MOOSE) guidelines [24] and the Preferred Reporting Items for Systematic Reviews and Meta-analyses (PRISMA) guidelines for manuscript reporting [25].

For the IPD meta-analysis, we included three studies providing non-identifiable IPD from the 17 studies included in the systematic review, comprising a total of 956 participants. Corresponding authors of all 17 studies were contacted to request IPD. IPD variables were analyzed largely as provided by the original studies. Categorical variables (e.g., sex and comorbidities) were recoded using consistent binary definitions where necessary to enable pooled analyses; no additional harmonization of continuous variables or outcome definitions was performed. Analyses of unadjusted pooled means included all participants with available cardiac outcome measures. For analyses adjusted for covariates, complete-case data were used.

**Search strategy.** The literature search was conducted across seven databases: Medline, PubMed, Embase (Ovid), Web of Science, CINAHL, Scopus, and PsycINFO. The search strategy used the PECO framework to ensure the identification of all relevant articles [26]. The Population included COVID-19 survivors with PASC; Exposure was COVID-19 infection; Comparators were healthy individuals, individuals previously hospitalized with COVID-19, and age- and gender-matched individuals without COVID-19; and Outcomes focused on cardiac function and structural measures. Initial searches and data extraction were conducted in August 2024 and were updated in October 2025 to include articles published up to that date. 3580 studies were identified across seven databases: PubMed (47), Embase (915), Scopus (2353), Web of Science (67), Medline Ovid (46), CINAHL (146), and PsycINFO (6). Detailed search strategies and results are in **Supplementary Tables 1–7.**

**Eligibility criteria.** Articles were eligible if they met the following criteria: (1) included adults (≥18 years old); and (2) assessed cardiac function and structure using non-invasive approaches such as echocardiography or CMR imaging. In this systematic review, cardiac function was evaluated in terms of systolic and diastolic performance. Systolic function was assessed using left ventricular ejection fraction (LVEF), Left Ventricular (LV) myocardial deformation (GLS), and right ventricular ejection fraction (RVEF), based on guidelines from the American Society of Echocardiography (ASE) and the European Association of Cardiovascular Imaging (EACVI) [13,14,27,28]. Diastolic function was assessed using the E/e' ratio, while right ventricular function was evaluated through tricuspid annular plane systolic excursion (TAPSE) and systolic pulmonary arterial pressure (sPAP). Cardiac structure, including chamber size, ventricular volumes, and wall thickness, was measured, with left ventricular end-diastolic volume (LVEDV) as a key indicator. Systolic and diastolic blood pressure (SBP and DBP) were also included as markers of cardiac workload. We excluded studies involving pregnant participants, those with a follow-up period shorter than 28 days, and review articles or studies that were not primary research.

**Fig. 1:**
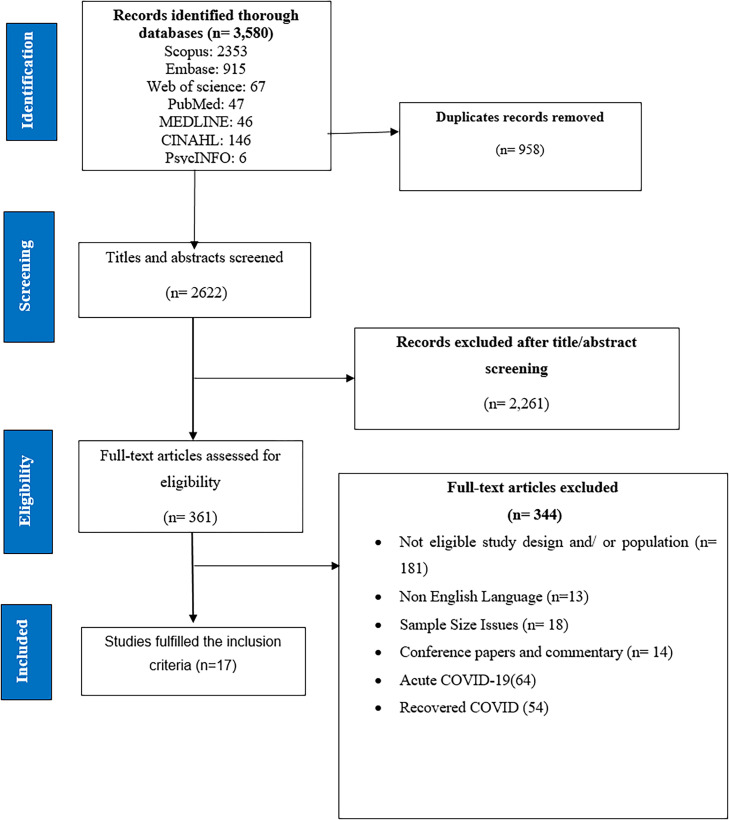
Flowchart depicting study selection. A total of 3580 records were identified from seven databases. After applying the exclusion criteria, 17 studies were included in the final analysis. Some studies reported data for more than one of the review outcomes of cardiac function/structure.

The search results were imported into Covidence, a web-based platform for managing systematic reviews **(Covidence, Melbourne, AU).** The selection process followed the four-step procedure (identification, screening, eligibility, and inclusion) described in the PRISMA guidelines [25] and is illustrated in Fig. 1. Titles, abstracts, and full texts were independently reviewed by two reviewers (BTS and LM), with unresolved discrepancies referred to a third reviewer.

**Data extraction.** The following information was extracted from studies: authors, publication year, population characteristics (including sample size and demographics), clinical characteristics (such as COVID-19 severity and comorbidities), cardiac function outcomes (including systolic and diastolic performance), cardiac structure outcomes (including ventricular size and systemic hemodynamic parameters), imaging modalities used, study location, follow-up period, study design, and effect sizes.

**Quality assessment.** We evaluated the quality of included studies using the Newcastle-Ottawa Scale (NOS) for cohort and case-control designs [29], and the Joanna Briggs Institute (JBI) tool for cross-sectional studies [30]. The NOS scores studies from 0 to 9, with 7 to 9 indicating high quality, 4 to 6 moderate quality, and 0 to 3 low quality. The JBI tool scores studies from 0 to 10, with 8 to 10 indicating high quality, 5 to 7 moderate quality, and 0 to 4 low quality. To assess publication bias, we used funnel plot visual inspection, Egger’s regression test, and the trim-and-fill method [31]. We also applied the Grading of Recommendations, Assessment, Development, and Evaluations (GRADE) criteria to evaluate the overall evidence quality [32,33], considering study design, risk of bias, consistency, directness, precision, and publication bias. The quality of the evidence was classified as very low, low, moderate, and high.

**Outcomes.** The meta-analysis included the following outcomes based on the available statistics reported by each study: LVEF, LVEDV, Left Ventricular Mass Index(LVMI), Left Ventricular Internal Dimension in Diastole(LVIDd), Left Ventricular End-Diastolic Volume Index(LVEDVi), Left Ventricular End-Systolic Volume Index(LVESVi), Left Atrial Volume Index(LAVI), the E/e' ratio, LV-GLS, RVEF, TAPSE, and sPAP in mmHg. Additionally, heart rate (beats per minute), SBP, and DBP for both the PASC and control groups were explored.

**Statistical analysis.** We employed a random-effects meta-analysis with the REML (Restricted Maximum Likelihood) method to assess the effect of PASC on cardiac structure and function outcomes, estimating pooled Mean Differences (MDs) with 95 % confidence intervals (CIs). Heterogeneity was evaluated with the I² statistic, where values of 25 %, 50 %, and 75 % indicated low, moderate, and high heterogeneity, respectively. Tau² was also estimated to measure variability in true effect sizes. To further investigate sources of heterogeneity, we conducted stratified analyses by age, sex, clinical status, and comorbidities. Cardiac function was analyzed as continuous variables (GLS and LVEF) and using categorical thresholds (GLS <16 %, LVEF <50 %) to assess the prevalence of subclinical dysfunction. We also performed a multiple linear regression analysis using IPD to assess the associations between predictors (HTN, DM, weight, height, age, BMI, and sex) and cardiovascular measures, including LVEF, RVEF, LVEDV, RVEDV, and LV-GLS.

In this study, we assessed potential publication bias using the trim-and-fill method, which estimates the number of missing studies, adjusts the overall effect size, and provides a more accurate estimate by accounting for potentially unpublished studies [34]. We also performed a sensitivity analysis through Leave-One-Out methodology to evaluate the robustness of our findings [35]. Meta-analyses and statistical testing were performed using R-Studio statistical software, with a two-tailed alpha level set at 0.05.

## 3 Results

**Study Selection and Characteristics.** Our systematic literature search identified 3580 records, of which 958 were duplicates. Initial screening based on titles resulted in 2072 articles. These articles were then screened by the abstracts to determine if they focused on the association of PASC with cardiac function and structure, which led to the exclusion of 1711 papers. At the eligibility stage, the full texts of 361 papers were reviewed, and 344 were excluded for not meeting the inclusion criteria. Seventeen studies with 4852 participants were included, of which 3932 contributed to the meta-analysis. IPD were requested from these studies, and data were obtained from three. The literature search process follows the PRISMA Guideline and is summarized using a flowchart as illustrated in Fig. 1.

**Supplementary Table 8** summarizes the baseline characteristics of the 17 studies included in the systematic review, comprising four cross-sectional studies, eleven cohort studies, and two case–control studies. Sample sizes ranged from 30 to 1154 participants. The mean age of participants varied between 51 and 60 years, and follow-up durations ranged from 6 weeks to 18 months, with six studies following participants for over a year. Control groups consisted of healthy participants, previously hospitalized COVID-19 survivors, and age- and sex-matched participants without a history of COVID-19. Transthoracic echocardiography (TTE) was the most commonly used imaging modality, followed by CMR. Detailed descriptions of cardiac imaging methods are provided in **Supplementary Material**, Section 4**.** Definitions of PASC varied across studies with respect to symptom duration, symptom domains, and ascertainment methods, and are summarized in **Supplementary Table 9**. Full citations for all included studies are available in the **Supplementary Material (page 18).**

**Left Ventricular Function.** In individuals with PASC, left ventricular function appears to be modestly impaired. The association between PASC and cardiac function was most apparent when assessed using LV-GLS and LVEF (see Table 1).

**Table 1:** Cardiac function and comorbidity prevalence in long COVID and control groups: pooled systematic review.

Characteristics	Pooled Mean (95 % CI)		Mean Difference (95 % CI)	P-value
	Long COVID-19 (*n* = 726)	Control (*n* = 230)		
**Demographic**				
Female **Sex**	522(71.9 %)	139(63.2 %)	–	0.000
**Age**	47.6(46.78–48.44)	65.55(63.6–66.7)	−17.5(−19.3- −15.7)	0.000
**Cardiac parameters**				
LVGLS	15.49(15.28–15.72)	19.13(18.85–19.4)	−3.63 (−4.74 to −2.92)	0.000
LVEF	61.36(60.70–62.02)	59.7(59.39–60.2)	1.577(0.33 - 2.83)	0.000
LVEDV	148.31(145.11–151.49)	163.27(149.1–177.4)	−14.96 (−30.34 to 0.42)	0.104
RVEDV	15.49(15.27–15.72)	26.82(22.21–31.44)	−11.33 (−18.02 to −4.64)	0.000
E’e average	7.64(7.31–7.98)	7.79(7.58–7.98)	−0.15 (−0.31 to 0.01)	0.226
LAVI	28.85(27.54–30.16)	29.81(28.91–30.69)	−0.96 (−2.74 to 0.82)	0.110
LVMI	70.52(68.1–72.9)	65.9(64.47–67.5)	4.62 (1.28 to 7.96)	0.999
LVIDd	4.51(4.42–4.6)	4.3(4.21–4.35)	0.21 (−0.01 to 0.43)	0.999
LVEDV2D Index	45.48(43.65–47.32)	43.5(42.87–44.26)	1.98(−0.61–4.45)	0.977
LVESV2D Index	17.62(16.71–18.54)	16.84(16.47–17.22)	0.78(−0.51–2.07)	0.947
**Comorbidity status**				
Diabetes	0.037(0.022–0.051)	0.29(0.24–0.35)	−0.25(−0.32- −0.19)	0.000
Hypertension	0.11(0.09–0.14)	0.65(0.59–0.71)	−0.53(−0.60- −0.47)	0.000

**CI:** Confidence Interval**| LVEF:** Left Ventricular Ejection Fraction | **LVGLS:** Left Ventricular Global Longitudinal Strain | **LVEDV:** Left Ventricular End-Diastolic Volume | **RVEDV:** Right Ventricular End-Diastolic Volume | **E’e average:** Average Early Diastolic Velocity of the Left Ventricle (E' wave) | **LAVI:** Left Atrial Volume Index | **LVMI:** Left Ventricular Mass Index | **LVIDd:** Left Ventricular Internal Diastolic Dimension| **LVEDV2D Index:** Left Ventricular End-Diastolic Volume 2-Dimensional Index| **LVESV2D Index:** Left Ventricular End-Systolic Volume 2-Dimensional Index.

**Fig. 2:**
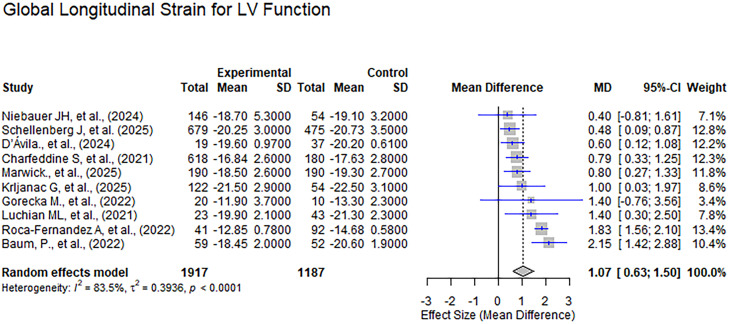
Forest plots of mean difference in Global Longitudinal Strain for LV Function.

In the pooled IPD analysis, the PASC group was associated with a significant reduction in GLS. The mean GLS was −15 % in the PASC group, compared to −19 % in the control group, indicating impaired myocardial deformation among individuals with PASC. Using categorical cut-offs, a higher proportion of individuals with PASC had abnormal GLS (<16 %) compared to controls (58.2 %, 95 % CI: 54.5–61.8 vs. 12.2 %, 95 % CI: 8.5–17.1) (Table 2). In line with the IPD findings, a meta-analysis of 10 studies showed that, compared to the control groups, LV-GLS was significantly reduced in the PASC group, with a mean difference of 1.07 % (95 % CI: 0.64 to 1.51; Fig. 2). A subsequent meta-regression indicated varying levels of LV-GLS across groups, with diabetes significantly contributing to the observed heterogeneity **(Supplementary Table 10).** Furthermore, IPD-based meta-regression revealed that individuals with hypertension and diabetes tend to exhibit worse LV-GLS **(Supplementary Table 11).** These findings are summarized in **Supplementary Table 12**, which provides a narrative interpretation of GLS changes as being associated with subclinical systolic dysfunction in the PASC group.

**Table 2:** Cardiac function parameters in control and Long COVID participants: an IPD analysis.

Measure	Category	Control ( %)	Long COVID ( %)
Global Longitudinal Strain(GLS)	GLS ≥16	87.8 %(82.9–91.4)	41.7 %(38.1–45.4)
	GLS <16	12.2 %(8.5–17.1)	58.2(54.5–61.8)
Ejection Fraction (EF)	EF ≥ 50 %	100 %	96.8 %(95.1–97.8)
	EF < 50 %	0 %	3.2 %(2.1–4.8)

No EF <50 % events observed in the control group; confidence interval not estimable.

**Fig. 3:**
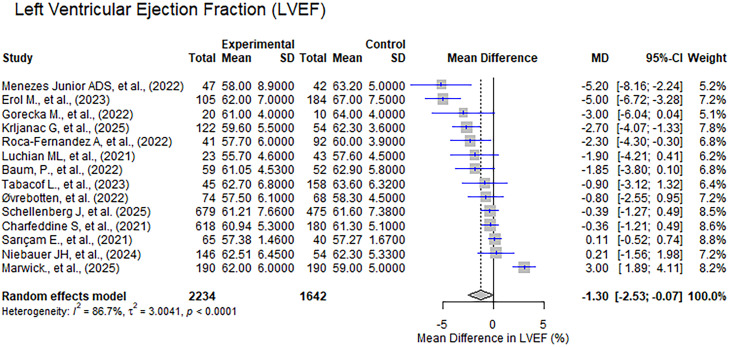
Forest plots of mean difference in Left Ventricular Ejection Fraction (LVEF).

In contrast, findings related to LVEF were more variable. IPD analysis showed that the PASC group was associated with a slightly higher mean LVEF (61 %) compared to controls (60 %) (Table 1). However, a post hoc analysis excluding the Australian cohort showed lower LVEF in the PASC group compared to controls, reversing the direction of the effect seen in the full IPD sample **(Supplementary Table 13).** The post hoc findings are in line with the meta-analysis results, which demonstrated a significant association between PASC and reduced LVEF (Fig. 3). Fourteen studies including 3876 participants (2234 PASC, 1642 controls) were analyzed. The random-effects pooled estimate showed that PASC was associated with a significant reduction in LVEF (MD = −1.30 %, 95 % CI: −2.53 to −0.07), with significant heterogeneity (τ² = 3.0; *p* < 0.001; I² = 86.7 %). Using categorical thresholds, the majority of participants had preserved LVEF (>50 %), with 3.2 % of individuals with PASC showing LVEF <50 %, compared to 0 % of controls (Table 2), supporting the overall finding of modest impairment in systolic function. Meta-regression indicated that older age and diabetes mellitus were associated with further reduction in LVEF **(Supplementary Table 10).** Additionally, the IPD analysis indicated that diabetes was associated with a reduction in LVEF among individuals with severe PASC **(Supplementary Table 11)**.

**Structural Cardiac Parameters.** Structural cardiac differences between individuals with and without PASC were generally modest and mostly non-significant. This section examines the relationships between PASC and cardiac structure using parameters such as LVIDd, LVMI, LVEDVi, LVESVi, E’e avg, LAVI, and LVEDV (Table 1). LVIDd was slightly higher in the Long COVID group (4.5 cm) than in controls (4.3 cm), with a non-significant mean difference of 0.2 cm. Likewise, the Long COVID group showed a marginally elevated LVMI (71 mg/m²) compared to controls (66 mg/m²), though this difference was not statistically significant. No meaningful differences were observed for LVEDVi and LVESVi, with mean values of 45 and 18 mL/m² respectively, in the Long COVID group, compared to 44 and 17 mL/m² in the controls. Furthermore, diastolic function measures, including E’e avg and LAVI, showed no notable differences.

**Fig. 4:**
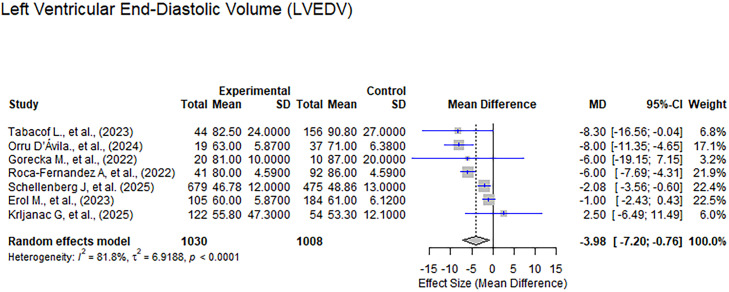
Forest plots of mean difference in Left Ventricular End-Diastolic Volume (LVEDV).

IPD analysis indicated a lower LVEDV in the Long COVID group (148 mL) compared to controls (163 mL), though the difference was not statistically significant (Table 1). However, this trend is supported by a random-effects meta-analysis, which showed a significant reduction in LVEDV among the PASC group compared to controls (MD = −3.98 mL, 95 % CI: −7.20 to −0.76), with substantial heterogeneity (τ² = 6.9; *p* < 0.001; I² = 81 %; Fig. 4). Meta-regression analysis identified older age as significantly associated with reduced LVEDV (*B* = −0.44 mL, **Supplementary Table 10**). Consistent with this, the IPD analysis also found a negative association between age and LVEDV **(Supplementary Table 11).**

**Right Ventricular Function and Diastolic Parameters.** The association between PASC and Right Ventricular Function and Diastolic Parameters was assessed using TAPSE, E/e' ratio, sPAP, RVEF, blood pressure, and heart rate (see Table 1).

**Fig. 5:**
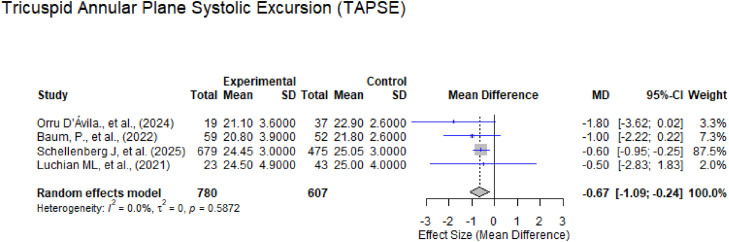
Forest Plot of Mean Differences in Tricuspid Annular Plane Systolic Excursion (TAPSE).

PASC was associated with lower TAPSE (MD = −0.67, 95 % CI: −1.09 to −0.24; *p* = 0.05) but with borderline statistical significance (Fig. 5). No heterogeneity was observed, and meta-regression did not identify any significant predictors **(Supplementary Table 10).** There were no significant differences in right heart parameters and diastolic markers due to PASC. Specifically, the E/e′ ratio showed no difference (MD = 0.00; *p* = 0.99), while the differences in RVEF and sPAP demonstrated borderline significance, with p-values of 0.08 and 0.29, respectively. Similarly, the mean difference in heart rate was 4.23 beats per minute (*p* = 0.19). There were small differences in systolic (−1.20 mmHg, *p* = 0.86) and diastolic blood pressure (−1.54 mmHg, *p* = 0.73), **Supplementary Figures 1–6.**

**Diabetes and Hypertension in Long COVID.** Diabetes and hypertension were reported in only 3.7 % and 11 % of Long COVID participants, versus 29 % and 65 % in controls, indicating a higher comorbidity burden in the control group (Table 1). To clarify their role, we conducted subgroup analyses focused on diabetes and hypertension. Findings show variations in LVGLS and LVEF by diabetes status **(Supplementary Table 14).** LVGLS was 19.4 % in controls without diabetes (reference), 18.7 % with diabetes (*p* = 0.009), 15.9 % in Long COVID participants with diabetes (*p* = 0.002), and 15.0 % without diabetes (*p* = 0.002), suggesting a more pronounced effect of Long COVID. LVEF was 61.9 % in controls without diabetes, 60.3 % with diabetes (*p* = 0.030), 58.9 % in Long COVID with diabetes (*p* = 0.232), and 59.5 % without (*p* < 0.001).

Similarly, in the hypertension analysis **(Supplementary Table 15),** LVGLS was 18.4 % in controls without hypertension and 19.4 % with hypertension. In Long COVID participants, LVGLS dropped to 15.1 % regardless of hypertension status. LVEF remained 61.4 % in both control subgroups, while Long COVID participants showed lower values: 59.7 % (with hypertension) and 59.4 % (without). Effect modification analysis showed a significant association between Long COVID and both LVEF (−2.69) and GLS (−4.48), indicating cardiac impairment **(Supplementary Table 16).** Overall, the results showed that while LVGLS and LVEF altered in individuals with hypertension and diabetes, the effects were minimal, with Long COVID having a more profound effect. IPD has a limited sample size; thus, these exploratory findings should be interpreted cautiously.

**Quality Assessment, Publication Bias, and Sensitivity Analyses.** All studies were assessed for quality using the NOS for case-control and cohort studies and the JBI risk of bias tool for cross-sectional studies. Among the studies, 9(64 %) were assessed as good quality, 5(36 %) as fair quality, and none were classified as poor quality **Supplementary Figures 7 and 8.** We also assessed publication bias for estimates of LVEF, LVEDV, LVGLS, TAPSE, and sPAP in individuals with PASC and controls. Visual inspection of funnel plots indicated no publication bias for studies reporting all these estimates **(Supplementary Figures 9–13)**. Additionally, Egger’s test yielded non-significant p-values for all parameters. For all parameters (LVEF, LVEDV, LVGLS, TAPSE, and sPAP), the p-values were well above 0.05 **(Supplementary Table 17).** A trim-and-fill analysis also found no missing studies **(Supplementary Figures 14–16).** However, as per Cochrane guidelines [36], tests for publication bias, such as funnel plots and Egger’s test, have limited reliability when fewer than 10 studies are included in a meta-analysis. Therefore, the results of these analyses should be interpreted with caution. Despite these limitations, the consistent findings across multiple methods provide no strong indication of publication bias, though its presence cannot be entirely ruled out.

The robustness of the results was further confirmed using a Leave-One-Out sensitivity analysis. The pooled MD for LVEF, LVEDV, and LV-GLS remained significant across all sensitivity scenarios. Results showed that removing individual studies had minimal impact on the overall estimates, indicating that the findings were robust. However, the heterogeneity remained high **(Supplementary Figures 17, 18, and 19).**

**In terms of overall evidence quality,** we included 17 observational studies in our review, all graded as low-certainty evidence according to GRADE. However, the risk of bias was low, and the studies were ranked as high quality based on several quality assessments **(Supplementary Table 18).** Regarding inconsistency, subgroup analysis, meta-regression, and sensitivity analysis addressed substantial heterogeneity, with results remaining moderately consistent, further supported by IPD analysis. The studies examined the direct effects of PASC on cardiac parameters; however, PASC definitions varied, and control groups were heterogeneous. Consequently, directness was rated as moderate. Precision was also rated as moderate, due to small sample sizes despite relatively narrow confidence intervals. Finally, Egger’s test, funnel plots, and the trim-and-fill method showed no indication of publication bias, supporting a high level of confidence in this domain. Despite these consistent findings and low risk of bias, the overall certainty of the evidence remains low because all data are observational and heterogeneous.

## 4 Discussion

This review suggests that COVID-19 survivors experiencing PASC exhibit modest, largely subclinical impairments in cardiac function, primarily involving the left ventricle. We examined data from 4852 individuals, including 3173 with PASC and 1679 controls. Among those with PASC, a modest yet statistically significant reduction was observed in LV-GLS, LVEF, and LVEDV. Cardiac impairments were evident not only in individuals with acute COVID-19 [6,22] but also in those who had recovered from the virus [21,37]. Moreover, impairments in cardiac function and structure resemble those seen following other viral infections, such as SARS and MERS [[38], [39], [40], [41], [42]]. These ongoing, subclinical impairments could enhance susceptibility to further cardiovascular injury. This highlights the need for continued follow-up and research to clarify their long-term clinical relevance.

To further explain the observed heterogeneity among included studies, we conducted meta-regression and IPD analysis. Our findings indicate that reductions in cardiac function were more pronounced among individuals with pre-existing hypertension or diabetes, as well as in those with more severe PASC. These findings are broadly consistent with current hypotheses on cardiac dysfunction following COVID-19, which propose distinct yet overlapping mechanisms across the acute and post-acute phases. It has been demonstrated that pre-existing cardiometabolic risk factors may reduce cardiac functional capacity [43,44], thereby increasing vulnerability to subsequent stressors. This vulnerability may become evident during acute COVID-19, when myocardial injury can occur through direct viral effects, systemic inflammation, and microvascular thrombosis [6,7]. In addition, PASC-specific processes, including chronic low-grade inflammation, autonomic dysfunction, endothelial dysfunction, and other post-viral sequelae, may contribute to persistent or delayed impairments in cardiac function [45,46]. It is also possible that reverse causality plays a role, with a predisposition to these underlying conditions increasing the likelihood of developing PASC.

We also observed no statistically significant differences between PASC and control groups in the E/e' ratio, RVEF, sPAP, heart rate, systolic blood pressure, or diastolic blood pressure. The lack of a significant difference in these measures may be attributed to PASC’s particular effect on cardiac function, or to subtle effects that take longer to manifest. In contrast to acute COVID-19 infection, PASC often appears to be more associated with the left ventricle than the right, and changes in parameters like the E/e' ratio or RVEF may be less pronounced if cardiac effects are mild or subclinical [7]. sPAP and autonomic dysfunction affecting heart rate and blood pressure may also remain stable, especially if lung or vascular involvement is not severe [47]. In addition, lifestyle, medication use, socioeconomic status, and genetic predispositions may also affect these parameters. We believe future studies need to emphasize the multifactorial and heterogeneous nature of risk factors related to cardiac dysfunction to better understand these complex associations.

Given that approximately 10–20 % of COVID-19 survivors are expected to develop PASC, even modest reductions in cardiac function may translate into a meaningful burden of cardiovascular vulnerability at the population level. In this review, PASC was associated with small reductions in left ventricular function, most consistently reflected by changes in LV-GLS and, to a lesser extent, LVEF. These findings should be interpreted as indicators of cardiovascular vulnerability rather than markers of inevitable progression to overt heart failure. The observed impairments were more pronounced among individuals with multiple persistent symptoms, older age, and cardiometabolic comorbidities. Taken together, these patterns support a risk-stratified approach to cardiovascular surveillance, with targeted monitoring of higher-risk individuals rather than routine cardiac imaging for all patients with PASC. Further longitudinal studies are needed to clarify the persistence, progression, and clinical significance of these subclinical changes, linking imaging to symptoms, biomarkers, and clinical outcomes.

**Strengths and Limitations.** This review has several strengths, including a comprehensive search strategy across seven databases and the inclusion of IPD from three studies, which supplemented the meta-regression analysis and enhanced the robustness of our findings. Both analyses show a significant association between reduced cardiac function and the severity of PASC, as well as preexisting comorbidities, in the multivariable-adjusted pooled effect estimates. We also assessed the risk of bias in the included studies and evaluated the certainty of the evidence using the GRADE approach. However, the findings should be interpreted in light of several limitations. First, although statistically significant, the observed differences in GLS among individuals with PASC (3.6 % [95 % CI 2.9 to 4.7]) were modest and largely subclinical. However, categorical analysis revealed that the majority of participants (58 %) had abnormal GLS (<16 %) compared to 12 % of controls, indicating meaningful subclinical myocardial impairment. While these values are comparable to those reported following potentially cardiotoxic chemotherapy [48], corresponding reductions in LVEF were smaller, and the clinical significance of these changes remains uncertain. In this context, the “multiple hit” hypothesis of heart failure postulates that an initial myocardial injury could be worsened over time by other sources of myocardial injury and stress, including diabetes mellitus, hypertension, and obesity [18]. Second, the subgroup and meta-regression analyses, while informative, remain exploratory and are based on a limited IPD sample size, which restricts the strength of causal inference. In addition, residual confounding cannot be excluded, as control groups were heterogeneous and, in some studies, had a higher prevalence of cardiometabolic risk factors than the PASC cohorts, reflecting imbalances in baseline characteristics that may influence results. Consequently, the observed cardiac abnormalities may reflect underlying vulnerability among individuals predisposed to both persistent symptoms and myocardial dysfunction, rather than effects directly attributable to PASC. Finally, heterogeneity in study design (cohort, case–control, and cross-sectional), follow-up duration, symptom definitions, and differences in imaging methods may have influenced the assessment of cardiac structure and function and may contribute to between-study differences **(see Supplementary Table 9).** In addition, GLS measurements may vary according to imaging protocols and analysis software; however, vendor-specific information was not consistently reported. Furthermore, while no evidence of publication bias was detected using funnel plots, Egger’s test, or trim-and-fill analysis, the small number of studies per outcome limits the reliability of these methods, and reporting bias cannot be fully excluded.

## 5 Conclusion

The review shows that PASC (Long COVID) is associated with modest but significant impairments in cardiac function, mainly affecting left ventricular performance, while structural heart changes and right ventricular function remain largely unchanged. Diabetes and older age appear to have an association with the observed decline. This finding emphasizes that routine cardiac imaging for all COVID-19 survivors with PASC is unlikely to be beneficial; instead, tailored evaluation of high-risk populations should be prioritized. The results also highlight the need for further research to have a better understanding of vulnerable COVID-19 survivors' long-term cardiovascular health.

## Funding

This work is part of the PERCEIVE study (Persistent Cardiovascular Effects of COVID-19 Viral Infection) and was funded by the Medical Research Future Fund (MRFF) under Grant number 2005874, as part of the MRFF 2020 Rare Cancers Rare Diseases Unmet Need COVID-19 Grant Opportunity. BTS is supported by a Melbourne Research Scholarship, a Rowden White Scholarship, and the Baker Bright Spark Top-Up Scholarship. THM is supported by an Investigator grant (2008129) from the NHMRC.

## Data Availability Statement

NAll data supporting the findings of this study are included in the manuscript and its supplementary materials. The IPD used in this analysis were obtained from three studies that are part of the systematic review. These data were shared with the authors through data-sharing agreements and are not publicly available due to privacy and confidentiality restrictions. Requests to access the IPD should be directed to the corresponding authors of the original studies, as cited in the Supplementary materials.

## Relationships with industry

The authors have no relationships with industry relevant to this work.

## Author agreement: the American Journal of Preventive Cardiology

Manuscript Title: The Impact of Post-Acute Sequelae of COVID-19 on Cardiac Function and Structure: A Systematic Review and a Hybrid Individual Participant Data Meta-Analysis

As the submitting author, I certify on behalf of all authors that this manuscript is original, not previously published, and not under consideration elsewhere. All authors meet ICMJE authorship criteria, have contributed substantially, approved the final manuscript, and agree on the author order. All conflicts of interest have been disclosed. All authors accept responsibility for the accuracy and integrity of the data, analyses, and conclusions.

Submitting Author: Binyam Tariku Seboka, MPH

Baker Department of Cardiometabolic Health, University of Melbourne and Baker Heart and Diabetes Institute

Email: bseboka@baker.edu.au

## CRediT authorship contribution statement

**Binyam Tariku Seboka:** Writing – review & editing, Writing – original draft, Visualization, Validation, Software, Methodology, Investigation, Formal analysis, Data curation, Conceptualization. **Lillian Ma:** Writing – review & editing, Writing – original draft, Software, Data curation. **Dianna J. Magliano:** Writing – review & editing, Writing – original draft. **Stella Talic:** Writing – review & editing, Writing – original draft, Supervision. **Antonio da Silva Menezes Junior:** Writing – review & editing, Writing – original draft. **Alessandra Borlotti:** Writing – review & editing, Writing – original draft. **Helena Thomaides Brears:** Writing – review & editing, Writing – original draft. **Andrea Dennis:** Writing – review & editing, Writing – original draft. **Amitava Banerjee:** Writing – review & editing, Writing – original draft. **Thomas H. Marwick:** Writing – review & editing, Writing – original draft, Validation, Supervision, Methodology, Funding acquisition, Formal analysis, Conceptualization. **Quan Huynh:** Writing – review & editing, Writing – original draft, Supervision, Methodology, Funding acquisition, Formal analysis, Conceptualization.

## Declaration of competing interest

The authors declare the following financial interests/personal relationships which may be considered as potential competing interests:

Quan Huynh reports financial support was provided by Medical Research Future Fund (MRFF). Thomas H. Marwick reports financial support was provided by Medical Research Future Fund (MRFF). If there are other authors, they declare that they have no known competing financial interests or personal relationships that could have appeared to influence the work reported in this paper.

## Appendix Supplementary materials

Image, application 1

Image, application 2
