# Supplementary material for: The impact of post-acute sequelae of COVID-19 on cardiac function and structure: A systematic review and a hybrid individual participant data meta-analysis
Source: Am J Prev Cardiol. 2026 Jan 31;27:101457. doi: 10.1016/j.ajpc.2026.101457 (PMC13261193; doi:10.1016/j.ajpc.2026.101457)
Supplement: Supplementary file 2 [file mmc2.docx]

**Supplementary Materials**

**The Impact of Post-Acute Sequelae of COVID-19 on Cardiac Function and Structure: A Systematic Review and A Hybrid Individual Participant Data Meta-Analysis**

**Appendix 1: Supplementary Tables, Figures, and Methodological Details**

# **Section 1: Preamble**

This methods appendix provides supplementary materials supporting our study titled *“The Impact of Post-Acute Sequelae of COVID-19 on Cardiac Function and Structure: A Systematic Review and a Hybrid Individual Participant Data Meta-Analysis.”* The appendix is organized as follows:

- **Section 2:** Supplementary Tables (including Supplementary Table 8: Summary characteristics of studies included in the systematic review, followed immediately by the references of included studies)
- **Section 3:** Supplementary Figures
- **Section 4:** Cardiac Imaging Methodology
- **Section 5:** Supplementary Statistical Methodology

Detailed descriptions of the cardiac imaging techniques and statistical approaches employed in this study are provided in Sections 4 and 5, respectively. The references for the studies included in the systematic review are presented directly after Supplementary Table 8 to provide full citation details.

This supplementary material aims to enhance transparency and reproducibility of our analyses. We have documented the analytical procedures and data sources used to generate the findings presented, adhering to established guidelines for systematic reviews and meta-analyses.

**Contents**

[**Section 1: Preamble** 2](#_Toc219555759)

[**Section 2: Supplementary Tables** 4](#_Toc219555760)

[**Section 3: Supplementary Figures** 29](#_Toc219555761)

[**Section 4: Cardiac imaging methodology** 46](#_Toc219555762)

[**Section 5: Supplementary Statistical Methodology** 48](#_Toc219555763)

# **Section 2: Supplementary Tables**

Supplementary Table 1: Search strategy for PubMed: limited to all adult (19 plus years)" and observational study

Supplementary Table 2: Search strategy for Medline: Limited to all adult (19 plus years)" and English and observational study

Supplementary Table 3: Search strategy for EMBASE: Limited to human and english language and "remove medline records" and "remove preprint records" and embase and (adult <18 to 64 years> or aged <65+ years>

Supplementary Table 4: Search strategy for Web of Science: limited to English language and articles Filed to: Title

Supplementary Table 5: Search strategy for Scopus: limited to Human, Articles, English study

Supplementary Table 6: Search strategy for CINAHL: limited to English Language

Supplementary Table 7: Search strategy for PsycINFO: Not limited to anything

Supplementary Table 8: Summary characteristics of studies included in the systematic review

Supplementary Table 9. Definitions of Post-Acute Sequelae of SARS-CoV-2 Infection (PASC) across included studies

Supplementary Table 10: Summary of Meta-Regression Analysis for Factors Affecting Mean Difference of cardiac outcomes

Supplementary Table 11: Multivariate Regression Analysis of Cardiac Function Metrics

Supplementary Table 12: Summary of Cardiac Parameters in Individuals with PASC vs. Controls: Narrative Interpretation of Meta-Analysis and IPD Findings

Supplementary Table 13: Post-hoc analysis excluding Australian cohort

Supplementary Table 14: Cardiovascular Impact of Long COVID: Comparison of LVGLS and LVEF in Individuals with and without Diabetes

Supplementary Table 15: Cardiovascular Impact of Long COVID: Comparison of LVGLS and LVEF in Individuals with and without hypertension

Supplementary Table 16: Effect Modification Analysis of LVEF and GLS

Supplementary Table 17: Summary of egger test parameters across cardiac outcomes

Supplementary Table 18: Overall quality of evidence based on GRADE approach

**Note:** The literature search was initially conducted on **August 14, 2024**, using the following databases: **Medline, PubMed, Embase (Ovid), EBM (Ovid), Web of Science, CINAHL,** and **Scopus**, and was later updated to include studies published **through the end of October 2025.**

| **Supplementary Table 1: Search strategy for PubMed: limited to**  **all adult (19 plus years)" and observational study** | | | | |
| --- | --- | --- | --- | --- |
|  | **Command** | **Strategies and keywords** | **Result** | **Date** |
| 1 | **COVID-19** | ("coronavirus 19" OR " coronavirus disease 2019" OR " COVID 19 " OR " corona virus*" OR " SARSCoV2" OR " SARSCoV‐2" OR " SARS CoV‐2" OR " Severe Acute Respiratory Syndrome Coronavirus‐2 " OR " SARS‐coronavirus‐2" OR "2019‐ncov " OR "2019‐novel CoV " OR "Covid 2019" OR " COVID19" OR "coronavirus*" OR "severe acute respiratory syndrome" OR " Sars‐coronavirus2" OR " SARS‐like coronavirus " OR "severe acute respiratory syndrome" OR " SARS‐CoV2", OR " Long COVID" OR " Post-acute sequelae of SARS-CoV-2 infection (PASC)" OR " Post-COVID condition" OR " COVID-19 sequelae" ) | 6,187 | 14/08/2024 |
|  |  | **AND** |  |  |
| 2 | **Cardiac function** | ("cardiac function" OR "cardiac structure" OR "cardiac dimensions" OR " ventricular dimensions" OR " cardiac output " OR "cardiac index" OR " ejection fraction " OR " diastolic function" OR "systolic function" OR " atrial function" OR " Cardiac Strain" OR " heart function" OR " ventricular function" ) | 3,592 | 14/08/2024 |
|  |  | ((("coronavirus 19"[All Fields] OR "coronavirus disease 2019"[All Fields] OR "COVID 19"[All Fields] OR "corona virus*"[All Fields] OR "SARSCoV2"[All Fields] OR "SARSCoV-2"[All Fields] OR "SARS CoV-2"[All Fields] OR "Severe Acute Respiratory Syndrome Coronavirus-2"[All Fields] OR "SARS-coronavirus-2"[All Fields] OR "2019-ncov"[All Fields] OR "2019-novel CoV"[All Fields] OR "Covid 2019"[All Fields] OR "COVID19"[All Fields] OR "coronavirus*"[All Fields] OR "severe acute respiratory syndrome"[All Fields] OR "Sars-coronavirus2"[All Fields] OR "SARS-like coronavirus"[All Fields] OR "severe acute respiratory syndrome"[All Fields] OR "SARS-CoV2"[All Fields]) AND ()) OR "Long COVID"[All Fields] OR "post acute sequelae of sars cov 2 infection pasc"[All Fields] OR "Post-COVID condition"[All Fields] OR "COVID-19 sequelae"[All Fields]) AND (("cardiac function"[All Fields] OR "cardiac structure"[All Fields] OR "cardiac dimensions"[All Fields] OR "ventricular dimensions"[All Fields] OR "cardiac output"[All Fields] OR "cardiac index"[All Fields] OR "ejection fraction"[All Fields] OR "diastolic function"[All Fields] OR "systolic function"[All Fields] OR "atrial function"[All Fields] OR "Cardiac Strain"[All Fields] OR "heart function"[All Fields] OR "ventricular function"[All Fields]) AND ((observationalstudy[Filter]) AND (alladult[Filter])) |  |  |
|  | **Final** | **1 AND 2** | **47** | 14/08/2024 |

| **Supplementary Table 2: Search strategy for Medline: Limited to all adult (19 plus years)" and English and observational study** | | | | |
| --- | --- | --- | --- | --- |
|  | **Command** | **Strategies and keywords** | **Result** | **Date** |
| 1 | **COVID-19** | ("coronavirus 19" OR " coronavirus disease 2019" OR " COVID 19 " OR " corona virus*" OR " SARSCoV2" OR " SARSCoV‐2" OR " SARS CoV‐2" OR " Severe Acute Respiratory Syndrome Coronavirus‐2 " OR " SARS‐coronavirus‐2" OR "2019‐ncov " OR "2019‐novel CoV " OR "Covid 2019" OR " COVID19" OR "coronavirus*" OR "severe acute respiratory syndrome" OR " Sars‐coronavirus2" OR " SARS‐like coronavirus " OR "severe acute respiratory syndrome" OR " SARS‐CoV2", OR " Long COVID" OR " Post-acute sequelae of SARS-CoV-2 infection (PASC)" OR " Post-COVID condition" OR " COVID-19 sequelae" ) | 4759 | 14/08/2024 |
|  |  | **AND** |  |  |
| 2 | **Cardiac function** | ("cardiac function" OR "cardiac structure" OR "cardiac dimensions" OR " ventricular dimensions" OR " cardiac output " OR " cardiac index" OR " ejection fraction " OR " diastolic function" OR " systolic function" OR " atrial function" OR " Cardiac Strain" OR " heart function" OR " ventricular function" ) | 3508 | 14/08/2024 |
|  |  | (("coronavirus 19" or " coronavirus disease 2019" or " COVID 19 " or " corona virus*" or " SARSCoV2" or " SARSCoV‐2" or " SARS CoV‐2" or " Severe Acute Respiratory Syndrome Coronavirus‐2 " or " SARS‐coronavirus‐2" or "2019‐ncov " or "2019‐novel CoV " or "Covid 2019" or " COVID19" or "coronavirus*" or "severe acute respiratory syndrome" or " Sars‐coronavirus2" or " SARS‐like coronavirus " or "severe acute respiratory syndrome" or " SARS‐CoV2," or " Long COVID" or " Post-acute sequelae of SARS-CoV-2 infection (PASC)" or " Post-COVID condition" or " COVID-19 sequelae") and ("cardiac function" or "cardiac structure" or "cardiac dimensions" or " ventricular dimensions" or " cardiac output " or " cardiac index" or " ejection fraction " or " diastolic function" or " systolic function" or " atrial function" or " Cardiac Strain" or " heart function" or " ventricular function")).af. |  |  |
|  | **Final** | **1 AND 2** | **46** | 14/08/2024 |

| **Supplementary Table 3: Search strategy for EMBASE: Limited to human and english language and "remove medline records" and "remove preprint records" and embase and (adult <18 to 64 years> or aged <65+ years>** | | | | |
| --- | --- | --- | --- | --- |
|  | **Command** | **Strategies and keywords** | **Result** | **Date** |
| 1 | **COVID-19** | ("coronavirus 19" OR " coronavirus disease 2019" OR " COVID 19 " OR " corona virus*" OR " SARSCoV2" OR " SARSCoV‐2" OR " SARS CoV‐2" OR " Severe Acute Respiratory Syndrome Coronavirus‐2 " OR " SARS‐coronavirus‐2" OR "2019‐ncov " OR "2019‐novel CoV " OR "Covid 2019" OR " COVID19" OR "coronavirus*" OR "severe acute respiratory syndrome" OR " Sars‐coronavirus2" OR " SARS‐like coronavirus " OR "severe acute respiratory syndrome" OR " SARS‐CoV2", OR " Long COVID" OR " Post-acute sequelae of SARS-CoV-2 infection (PASC)" OR " Post-COVID condition" OR " COVID-19 sequelae" ) | 46218 | 14/08/2024 |
|  |  | **AND** |  |  |
| 2 | **Cardiac function** | ("cardiac function" OR "cardiac structure" OR "cardiac dimensions" OR " ventricular dimensions" OR " cardiac output " OR " cardiac index" OR " ejection fraction " OR " diastolic function" OR " systolic function" OR " atrial function" OR " Cardiac Strain" OR " heart function" OR " ventricular function" ) | 30768 | 14/08/2024 |
|  |  | ("coronavirus 19" or " coronavirus disease 2019" or " COVID 19 " or " corona virus*" or " SARSCoV2" or " SARSCoV‐2" or " SARS CoV‐2" or " Severe Acute Respiratory Syndrome Coronavirus‐2 " or " SARS‐coronavirus‐2" or "2019‐ncov " or "2019‐novel CoV " or "Covid 2019" or " COVID19" or "coronavirus*" or "severe acute respiratory syndrome" or " Sars‐coronavirus2" or " SARS‐like coronavirus " or "severe acute respiratory syndrome" or " SARS‐CoV2," or " Long COVID" or " Post-acute sequelae of SARS-CoV-2 infection (PASC)" or " Post-COVID condition" or " COVID-19 sequelae") AND ("cardiac function" or "cardiac structure" or "cardiac dimensions" or " ventricular dimensions" or " cardiac output " or " cardiac index" or " ejection fraction " or " diastolic function" or " systolic function" or " atrial function" or " Cardiac Strain" or " heart function" or " ventricular function").mp. [mp=title, abstract, heading word, drug trade name, original title, device manufacturer, drug manufacturer, device trade name, keyword heading word, floating subheading word, candidate term word] |  |  |
|  | **Final** | **1 AND 2** | **915** | 14/08/2024 |

| **Supplementary Table 4: Search strategy for Web of Science: limited to**  **English language and articles Filed to: Title** | | | | |
| --- | --- | --- | --- | --- |
|  | **Command** | **Strategies and keywords** | **Result** | **Date** |
| 1 | **COVID-19** | ("coronavirus 19" OR " coronavirus disease 2019" OR " COVID 19 " OR " corona virus*" OR " SARSCoV2" OR " SARSCoV‐2" OR " SARS CoV‐2" OR " Severe Acute Respiratory Syndrome Coronavirus‐2 " OR " SARS‐coronavirus‐2" OR "2019‐ncov " OR "2019‐novel CoV " OR "Covid 2019" OR " COVID19" OR "coronavirus*" OR "severe acute respiratory syndrome" OR " Sars‐coronavirus2" OR " SARS‐like coronavirus " OR "severe acute respiratory syndrome" OR " SARS‐CoV2", OR " Long COVID" OR " Post-acute sequelae of SARS-CoV-2 infection (PASC)" OR " Post-COVID condition" OR " COVID-19 sequelae" ) | 279,337 | 14/08/2024 |
|  |  | **AND** |  |  |
| 2 | **Cardiac function** | ("cardiac function" OR "cardiac structure" OR "cardiac dimensions" OR " ventricular dimensions" OR " cardiac output " OR " cardiac index" OR " ejection fraction " OR " diastolic function" OR " systolic function" OR " atrial function" OR " Cardiac Strain" OR " heart function" OR " ventricular function" ) | 31,884 | 14/08/2024 |
|  | **Final** | ("coronavirus 19" OR " coronavirus disease 2019" OR " COVID 19 " OR " corona virus*" OR " SARSCoV2" OR " SARSCoV‐2" OR " SARS CoV‐2" OR " Severe Acute Respiratory Syndrome Coronavirus‐2 " OR " SARS‐coronavirus‐2" OR "2019‐ncov " OR "2019‐novel CoV " OR "Covid 2019" OR " COVID19" OR "coronavirus*" OR "severe acute respiratory syndrome" OR " Sars‐coronavirus2" OR " SARS‐like coronavirus " OR "severe acute respiratory syndrome" OR " SARS‐CoV2", OR " Long COVID" OR " Post-acute sequelae of SARS-CoV-2 infection (PASC)" OR " Post-COVID condition" OR " COVID-19 sequelae" ) (Title) and English (Languages) and English (Languages) and Article (Document Types) AND("cardiac function" OR "cardiac structure" OR "cardiac dimensions" OR " ventricular dimensions" OR " cardiac output " OR " cardiac index" OR " ejection fraction " OR " diastolic function" OR " systolic function" OR " atrial function" OR " Cardiac Strain" OR " heart function" OR " ventricular function" ) (Title) and English (Languages) and English (Languages) and Article (Document Types) |  |  |
|  |  | **1 AND 2** | **63** | 14/08/2024 |

| **Supplementary Table 5: Search strategy for**  **Scopus : limited to**  **Human, Articles, English study** | | | | |
| --- | --- | --- | --- | --- |
|  | **Command** | **Strategies and keywords** | **Result** | **Date** |
| 1 | **COVID-19** | ("coronavirus 19" OR " coronavirus disease 2019" OR " COVID 19 " OR " corona virus*" OR " SARSCoV2" OR " SARSCoV‐2" OR " SARS CoV‐2" OR " Severe Acute Respiratory Syndrome Coronavirus‐2 " OR " SARS‐coronavirus‐2" OR "2019‐ncov " OR "2019‐novel CoV " OR "Covid 2019" OR " COVID19" OR "coronavirus*" OR "severe acute respiratory syndrome" OR " Sars‐coronavirus2" OR " SARS‐like coronavirus " OR "severe acute respiratory syndrome" OR " SARS‐CoV2", OR " Long COVID" OR " Post-acute sequelae of SARS-CoV-2 infection (PASC)" OR " Post-COVID condition" OR " COVID-19 sequelae" ) | 253,512 | 14/08/2024 |
|  |  | **AND** |  |  |
| 2 | **Cardiac function** | ("cardiac function" OR "cardiac structure" OR "cardiac dimensions" OR " ventricular dimensions" OR " cardiac output " OR " cardiac index" OR " ejection fraction " OR " diastolic function" OR " systolic function" OR " atrial function" OR " Cardiac Strain" OR " heart function" OR " ventricular function" ) | 198,691 | 14/08/2024 |
|  | **Final query** | ( TITLE-ABS-KEY ( ( "coronavirus 19" OR " coronavirus disease 2019" OR " COVID 19 " OR " corona virus*" OR " SARSCoV2" OR " SARSCoV‐2" OR " SARS CoV‐2" OR " Severe Acute Respiratory Syndrome Coronavirus‐2 " OR " SARS‐coronavirus‐2" OR "2019‐ncov " OR "2019‐novel CoV " OR "Covid 2019" OR " COVID19" OR "coronavirus*" OR "severe acute respiratory syndrome" OR " Sars‐coronavirus2" OR " SARS‐like coronavirus " OR "severe acute respiratory syndrome" OR " SARS‐CoV2" , OR " Long COVID" OR " Post-acute sequelae of SARS-CoV-2 infection (PASC)" OR " Post-COVID condition" OR " COVID-19 sequelae" ) ) ) AND ( TITLE-ABS-KEY ( ( "cardiac function" OR "cardiac structure" OR "cardiac dimensions" OR " ventricular dimensions" OR " cardiac output " OR " cardiac index" OR " ejection fraction " OR " diastolic function" OR " systolic function" OR " atrial function" OR " Cardiac Strain" OR " heart function" OR " ventricular function" ) ) ) AND ( LIMIT-TO ( LANGUAGE , "English" ) ) AND ( LIMIT-TO ( DOCTYPE , "ar" ) ) AND ( LIMIT-TO ( EXACTKEYWORD , "Human" ) ) |  |  |
|  | **Final** | **1 AND 2** | **2,353** | 14/08/2024 |

| **Supplementary Table 6: Search strategy for CINAHL: limited to**  **English Language** | | | | |
| --- | --- | --- | --- | --- |
|  | **Command** | **Strategies and keywords** | **Result** | **Date** |
| 1 | **COVID-19** | ("coronavirus 19" OR " coronavirus disease 2019" OR " COVID 19 " OR " corona virus*" OR " SARSCoV2" OR " SARSCoV‐2" OR " SARS CoV‐2" OR " Severe Acute Respiratory Syndrome Coronavirus‐2 " OR " SARS‐coronavirus‐2" OR "2019‐ncov " OR "2019‐novel CoV " OR "Covid 2019" OR " COVID19" OR "coronavirus*" OR "severe acute respiratory syndrome" OR " Sars‐coronavirus2" OR " SARS‐like coronavirus " OR "severe acute respiratory syndrome" OR " SARS‐CoV2", OR " Long COVID" OR " Post-acute sequelae of SARS-CoV-2 infection (PASC)" OR " Post-COVID condition" OR " COVID-19 sequelae" ) | 37,392 | 14/08/2024 |
|  |  | **AND** |  |  |
| 2 | **Cardiac function** | ("cardiac function" OR "cardiac structure" OR "cardiac dimensions" OR " ventricular dimensions" OR " cardiac output " OR " cardiac index" OR " ejection fraction " OR " diastolic function" OR " systolic function" OR " atrial function" OR " Cardiac Strain" OR " heart function" OR " ventricular function" ) | 56,729 | 14/08/2024 |
|  | **Final query** | ("coronavirus 19" OR " coronavirus disease 2019" OR " COVID 19 " OR " corona virus*" OR " SARSCoV2" OR " SARSCoV‐2" OR " SARS CoV‐2" OR " Severe Acute Respiratory Syndrome Coronavirus‐2 " OR " SARS‐coronavirus‐2" OR "2019‐ncov " OR "2019‐novel CoV " OR "Covid 2019" OR " COVID19" OR "coronavirus*" OR "severe acute respiratory syndrome" OR " Sars‐coronavirus2" OR " SARS‐like coronavirus " OR "severe acute respiratory syndrome" OR " SARS‐CoV2", OR " Long COVID" OR " Post-acute sequelae of SARS-CoV-2 infection (PASC)" OR " Post-COVID condition" OR " COVID-19 sequelae" ) AND ("cardiac function" OR "cardiac structure" OR "cardiac dimensions" OR " ventricular dimensions" OR " cardiac output " OR " cardiac index" OR " ejection fraction " OR " diastolic function" OR " systolic function" OR " atrial function" OR " Cardiac Strain" OR " heart function" OR " ventricular function" ) |  |  |
|  | **Final** | **1 AND 2** | **146** | 14/08/2024 |

| **Supplementary Table 7: Search strategy for**  **PsycINFO: Not limited to**  **anything** | | | | |
| --- | --- | --- | --- | --- |
|  | **Command** | **Strategies and keywords** | **Result** | **Date** |
| 1 | **COVID-19** | ("coronavirus 19" OR " coronavirus disease 2019" OR " COVID 19 " OR " corona virus*" OR " SARSCoV2" OR " SARSCoV‐2" OR " SARS CoV‐2" OR " Severe Acute Respiratory Syndrome Coronavirus‐2 " OR " SARS‐coronavirus‐2" OR "2019‐ncov " OR "2019‐novel CoV " OR "Covid 2019" OR " COVID19" OR "coronavirus*" OR "severe acute respiratory syndrome" OR " Sars‐coronavirus2" OR " SARS‐like coronavirus " OR "severe acute respiratory syndrome" OR " SARS‐CoV2", OR " Long COVID" OR " Post-acute sequelae of SARS-CoV-2 infection (PASC)" OR " Post-COVID condition" OR " COVID-19 sequelae" ) | 52279 | 14/08/2024 |
|  |  | **AND** |  |  |
| 2 | **Cardiac function** | ("cardiac function" OR "cardiac structure" OR "cardiac dimensions" OR " ventricular dimensions" OR " cardiac output " OR " cardiac index" OR " ejection fraction " OR " diastolic function" OR " systolic function" OR " atrial function" OR " Cardiac Strain" OR " heart function" OR " ventricular function" ) | 2443 | 14/08/2024 |
|  | **Final query** | ("coronavirus 19" or " coronavirus disease 2019" or " COVID 19 " or " corona virus*" or " SARSCoV2" or " SARSCoV‐2" or " SARS CoV‐2" or " Severe Acute Respiratory Syndrome Coronavirus‐2 " or " SARS‐coronavirus‐2" or "2019‐ncov " or "2019‐novel CoV " or "Covid 2019" or " COVID19" or "coronavirus*" or "severe acute respiratory syndrome" or " Sars‐coronavirus2" or " SARS‐like coronavirus " or "severe acute respiratory syndrome" or " SARS‐CoV2," or " Long COVID" or " Post-acute sequelae of SARS-CoV-2 infection (PASC)" or " Post-COVID condition" or " COVID-19 sequelae").mp. [mp=title, abstract, heading word, table of contents, key concepts, original title, tests & measures, mesh word] AND |  |  |
|  | **Final** | **1 AND 2** | **6** | 14/08/2024 |

| **Supplementary Table 8: Summary characteristics of studies included in the systematic review** | | | | | | | | | |
| --- | --- | --- | --- | --- | --- | --- | --- | --- | --- |
| **Authors & year** | **Study design** | **Sample size** | **Country** | **Group (gender:%M)** | **Mean age (SD), years** | **Post‐COVID**  **follow‐up** | **Cardiac assessment method** | **Study period** | **Control group** |
| Luchian et al., (2021) | Cohort | 66 | Belgium | L: 14(60.9)  C:31(72.1) | L:51.3+ 11.3  C:51.6+11.1 | One year | Echo | March to April 2020 | Recovered healthy controls |
| Tauekelova et al., (2023) | Cohort | 312 | Kazakhstan | 111(35.6) | 54(42.5, 60) | 6 month | Echo | January 2021 | No control |
| Baum et al., (2022) | Cohort | 227 | Germany | L:20(NR)  C:198 | L:55.2+ 15.6  C: 48 + 14.34 | 3 to 18 months | Echo | April to December 2021 | Recovered healthy controls |
| Erol et al., (2023) | Case-control | 289 | Turkey | L:45(42.8)  C:83(45.1) | L:56.1+ 11.3  C: 55.8+ 10.7 | At least a year | SPECT | December 2021 to December 2022 | Age and gender-matched individuals without a history of COVID-19 |
| Gorecka M., et al., (2022) | Case-control | 30 | United Kingdom | L:9(47)  C: 6(60) | L:45+ 13  C:51+11 | 12 weeks | CMR | March  2021 to July 2021 | Healthy Individuals |
| Charfeddine et al., (2021) | Cross-sectional | 798 | Tunisia | L:229(37.1)  C:86(47.8) | L: 50.03+ 14.2  C: 49.65+14.3 | >= 4 weeks | Echo | January 20 t0 May 10, 2021 | Recovered healthy controls |
| Roca-Fernandez et al., (2023) | Cohort | 534 | United Kingdom | L:147(28)  C:NR | L:44(38-52)  C:NR | at six month and 12 month | CMR | April 2020 to October 2021 | Age and gender-matched Healthy individuals |
| Niebauer et al., (2024) | Cohort | 200 | Austria | L:81(55)  C:34(63) | L: 54.3+ 13.9  C: 50.78 + 14.1 | at six month and 18 month | Echo | February 2020 to October 2021 | Previously hospitalized COVID-19 patients |
| Menezes-Junior et al., (2022) | Cross-sectional | 89 | Brazil | L:19(40.4)  C:12(28.6) | L:44.4+ 12.2  C: 39.6 + 12.9 | At least 3 months after infection | Echo | August 2020 to June 2021 | Healthy Individuals |
| Tabacof et al., (2023) | Cohort | 203 | USA | L:68(33) | L: 45(22-80) | Four or more weeks | Echo | NR; Not Reported | No controls |
| Sarıçam et al., (2021) | Cross-sectional | 105 | Turkey | L: 35(70)  C: 15(30) | L: 37.35+ 7.01  C: 38.43+ 7.21 | Up to 2 months | Echo | December 2020 to  February 2021 | Recovered healthy controls |
| Øvrebotten et al., (2022). | Cohort | 142 | Norway | L: 74  C: 68 | 58.2 ± 13.5 | Up to 12 months | Echo | February 26 to June 29, 2021 | Individuals with a history of COVID |
| Marwick., et al., (2025) | Cohort | 380 | Australia | L:30(15.8)  C: 30(15.8) | L: 57.45+ 5.80  C: 57.45+ 5.80 | 3.2 to 12.6 months | Echo | NA | Pre-COVID matched controls |
| D’Ávila et al., 2024) | Cross-sectional | 56 | Brazil | L:17(46)  C:6(32) | L: 53+ 11  C: 54+ 6 | 7.9 months | Echo | June 2021 to  August 2022 | Individuals with a history of COVID |
| Schellenberg et al., (2025) | Cohort | 1154 | Germany | L:446(66)  C:314(66) | L:49+12  C: 48 + 12 | 17.2 months | Echo | October 1, 2020, to April 1, 2021 | age- and sex-matched |
| Krljanac et al., (2025) | Cohort | 176 | Serbia | L:53(43.4)  C:20(37) | L: 55.89 + 12.24  C: 56.07 + 12.41 | 6.2 ± 2.7 months | Echo, CMR | January to July 2021 | Individuals with a history of COVID |
| Trivieri et al., (2025) | Cohort | 91 | USA | L:53%  C:56% | L:48.5  C:42 | 9–12 months | 18F-FDG PET/MRI | December 2020 to July 2021 | Individuals with a history of COVID |
|  |  |  |  |  |  |  |  |  |  |

**Supplementary References**

**S1. Luchian ML**, et al. (2021). Subclinical myocardial dysfunction in patients with persistent dyspnea one year after COVID-19. *Diagnostics (Basel)*. 2021;12(1).

**S2. Tauekelova AT**, et al. (2023). Association of lung fibrotic changes and cardiological dysfunction with comorbidities in long COVID-19 cohort. *Int J Environ Res Public Health*. 2023;20(3).

**S3. Baum P**, et al. (2022). Cardiac function in relation to functional status and fatigue in patients with post-COVID syndrome. *Sci Rep*. 2022;12(1):19575.

**S4. Erol M**, et al. (2023). The role of myocardial perfusion imaging in predicting myocardial ischemia in patients diagnosed with long COVID. *Int J Cardiovasc Imaging*. 2023;39(11):2279–84.

**S5. Gorecka M**, et al. (2022). Cardiovascular magnetic resonance imaging and spectroscopy in clinical long-COVID-19 syndrome: A prospective case-control study. *J Cardiovasc Magn Reson*. 2022;24(1):50.

**S6. Charfeddine S**, et al. (2021). Long COVID-19 syndrome: Is it related to microcirculation and endothelial dysfunction? Insights from TUN-EndCOV study. *Front Cardiovasc Med*. 2021;8:745758.

**S7. Roca-Fernandez A**, et al. (2023). Cardiac abnormalities in long COVID 1-year post-SARS-CoV-2 infection. *Open Heart*. 2023;10(1).

**S8. Niebauer JH**, et al. (2024). Severe COVID-19 and its cardiopulmonary effects 6 and 18 months after hospital discharge. *Front Cardiovasc Med*. 2024;11:1366269.

**S9. Menezes Junior ADS**, et al. (2022). Cardiac autonomic function in long COVID-19 using heart rate variability: An observational cross-sectional study. *J Clin Med*. 2022;12(1).

**S10. Tabacof L**, et al. (2023). Dysautonomia, but not cardiac dysfunction, is common in a cohort of individuals with long COVID. *J Pers Med*. 2023;13(11).

**S11. Sarıçam E**, et al. (2021). Laboratory and imaging evaluation of cardiac involvement in patients with post-acute COVID-19. *Int J Gen Med*. 2021;14:4977–85.

**S12. Øvrebotten**, et al. (2022). Changes in cardiac structure and function from 3 to 12 months after hospitalization for COVID-19. Clin Cardiol, 2022. 45(10): p. 1044-1052

**S13. Marwick TH**, et al. (2025). Cardiac function and functional capacity in patients with long COVID: A comparison to propensity-matched community controls. *J Am Soc Echocardiogr*. 2025;38(1):16–23.e1.

**S14. D'Ávila O**, et al. (2024). Longitudinal strain and myocardial work in symptomatic patients having recovered from COVID-19 and possible associations with the severity of the disease. *Int J Cardiovasc Imaging*. 2024;40(4):745–56.

**S15. Schellenberg J**, et al. (2025). Cardiac structure and function 1.5 years after COVID-19: results from the EPILOC study. Infection 53, 1685–1697 (2025).

**S16.** **Krljanac G**, et al., (2025). Cardiovascular Manifestations of Patients with Long COVID. Diagnostics 2025, 15, 1771. https://doi.org/10.3390/diagnostics15141771

**S17.** **Trivieri MG**. et al. (2025). Prevalence of Persistent Cardiovascular and Pulmonary Abnormalities on PET/MRI and DECT Imaging in Long COVID Patients. J Nucl Med. 2025 Jul 1;66(7):1126-1134

| **Supplementary Table 9. Definitions of Post-Acute Sequelae of SARS-CoV-2 Infection (PASC) across included studies** | | | | |
| --- | --- | --- | --- | --- |
| **Study (Author, Year)** | **Time since infection** | **Symptom requirement** | **Domains required** | **How PASC was operationalised** |
| Luchian et al., (2021) | 12 months | Persistent dyspnoea | Respiratory, cardiovascular | PCR-confirmed COVID-19; no prior cardiopulmonary disease; ; symptom questionnaires |
| Tauekelova et al., (2023) | ≥3 months (mean 5.8) | Any persistent symptom | Respiratory, cardiovascular, systemic, neurological, GI, dermatologic | PCR-confirmed COVID-19; symptom questionnaires |
| Baum et al., (2022) | ≥12 weeks (mean 7 months) | ≥1 persistent symptom | Systemic, respiratory, cardiovascular, neuropsychological | PCR-confirmed COVID-19; fatigue & psychological scales |
| Erol et al., (2023) | ≥12 months | Ongoing COVID-related complaints | Cardiovascular (ischemia-related) | PCR-confirmed COVID-19; excluded major comorbidities; ; symptom questionnaires |
| Gorecka M., et al., (2022) | ≥12 weeks | Persistent symptoms | Cardiovascular | PCR-confirmed COVID-19; NICE long-COVID criteria; no prior CVD |
| Charfeddine et al., (2021) | ≥4 weeks | Long-COVID symptoms | Systemic, cardiovascular, neurological | PCR-confirmed COVID-19; symptom-based long-COVID diagnosis |
| Roca-Fernandez et al., (2023) | 6 and 12 months | Persistent symptoms | Multiorgan including cardiovascular | PCR-confirmed COVID-19; symptom questionnaires |
| Niebauer et al., (2024) | 6 and 18 months | Persistent symptoms | Cardiopulmonary, cardiovascular, respiratory | PCR-confirmed COVID-19; long-COVID clinical criteria |
| Menezes-Junior et al., (2022) | ≥3 months | Persistent symptoms | Autonomic, cardiopulmonary | PCR-confirmed COVID-19; WHO long-COVID criteria |
| Tabacof et al., (2023) | ≥4 weeks | Persistent symptoms | Autonomic, cardiovascular | PCR-confirmed COVID-19; symptom questionnaires |
| Sarıçam et al., (2021) | Up to 8 weeks | Palpitations or exertional symptoms | Cardiovascular | PCR-confirmed COVID-19; symptom questionnaires |
| Øvrebotten et al., (2022). | 3 and 12 months | Dyspnoea or persistent symptoms | Cardiopulmonary | PCR-confirmed COVID-19; symptom questionnaires |
| Marwick., et al., (2025) | 6 and 12 months | Persistent symptoms | Functional, cardiovascular | PCR-confirmed COVID-19; ; symptom questionnaires |
| D’Ávila et al., 2024) | ≥3 months | Persistent symptoms | Cardiovascular | PCR-confirmed COVID-19; symptom questionnaires |
| Schellenberg et al., (2025) | 18 months | Persistent PCS | Cardiopulmonary | PCR-confirmed COVID-19; symptom questionnaires |
| Krljanac et al., (2025) | ≥12 weeks (mean 6 months) | Persistent exertional symptoms | Cardiovascular, systemic | PCR-confirmed COVID-19; excluded alternative causes; symptom questionnaires |
| Trivieri et al., (2025) | 9–12 months | Persistent cardiopulmonary symptoms | Cardiopulmonary, inflammatory | PCR-/serology-confirmed COVID-19; symptom questionnaires |
| Abbreviations: PASC = Post-Acute Sequelae of SARS-CoV-2 Infection; PCR = Polymerase Chain Reaction; GI = Gastrointestinal; PCFS = Post-COVID-19 Functional Status; NT-proBNP = N-terminal pro B-type Natriuretic Peptide; TTE = Transthoracic Echocardiography; CMR = Cardiac Magnetic Resonance; 31P-CMRS = Phosphorus-31 Cardiac Magnetic Resonance Spectroscopy; ECG = Electrocardiogram; Holter = 24-hour ECG monitoring; SPECT MPI = Single-Photon Emission Computed Tomography Myocardial Perfusion Imaging; PET = Positron Emission Tomography; CPET = Cardiopulmonary Exercise Testing; DECT = Dual-Energy Computed Tomography; PROs = Patient-Reported Outcomes. | | | | |

| **Supplementary Table 10: Summary of Meta-Regression Analysis for Factors Affecting Mean Difference of cardiac outcomes** | | | |
| --- | --- | --- | --- |
| **Covariate** | **Coefficient** | **95% CI** | **Number of Studies** |
| **Left Ventricular Ejection Fraction** |  |  |  |
| Hypertension | -0.391 | -1.324, 0.542 | 8 |
| Diabetes | -0.348 | -0.605, -0.090 | 10 |
| Coronary Heart Disease | 0.7390 | -1.380, 2.858 | 5 |
| Age | -0.665 | -0.883, -0.446 | 10 |
| Sex(Male) | -0.236 | -0.6467, -.1729 | 10 |
| ICU Admission | 0.008 | -0.149, 0.164 | 7 |
| **Left Ventricular End-Diastolic Volume** |  |  |  |
| Hypertension | 0.192 | -0.547 to 0.931 | 4 |
| Diabetes | 0.140 | -0.282 to 0.563 | 4 |
| Age | -0.441 | -0.611 to -0.271 | 6 |
| Sex(Male) | .0492 | -0.414, to0.512 | 6 |
| **Global Longitudinal Strain for LV Function** |  |  |  |
| Hypertension | -0.046 | -0.672 to 0.579 | 5 |
| Diabetes | -0.055 | -0.116 to -0.006 | 7 |
| Coronary Heart Disease | -0.629 | -3.263 to 2.005 | 5 |
| Age | -0.173 | -0.495 to 0.150 | 8 |
| Sex(Male) | 0.119 | -0.068 to 0.092 | 8 |
| ICU Admission | -0.031 | -0.173 to 0.112 | 6 |
| **Tricuspid Annular Plane Systolic Excursion(TAPSE)** |  |  |  |
| Hypertension | 0.043 | -0.236, 0.322 | 3 |
| Diabetes | 0.063 | -0.041, 0.168 | 4 |
| Age | 0.092 | -0.068, 0.251 | 4 |
| Sex(Male) | 0.283 | -0.008, 0.064 | 4 |
| ICU Admission | 0.017 | -0.074, 0.108 | 3 |

| **Supplementary Table 11: Multivariate Regression Analysis of Cardiac Function Metrics** | | | | | |
| --- | --- | --- | --- | --- | --- |
|  |  | **Mild long COVID** | | **Severe Long COVID** | |
| **Cardiac Metrics** | **Predictors** | **Coefficient[95% CI]** | **p-value** | **Coefficient[95% CI]** | **p-value** |
| **Left Ventricular Ejection Fraction (LVEF)** | hypertension | -0.95[-2.75, 0.86] | 0.304 | 0.60[-1.70, 2.90] | 0.608 |
|  | diabetes | -0.81[-5.71, 4.08] | 0.745 | -3.40[-6.94, -0.14] | 0.030 |
|  | weight | -0.17[-0.47, 0.12] | 0.253 | -0.07[-0.41, 0.27] | 0.698 |
|  | height | 0.07[-0.20, 0.33] | 0.624 | 0.06[-0.27, 0.40] | 0.700 |
|  | age | 0.06[0.02, 0.10] | 0.007 | 0.04[-0.03, 0.11] | 0.247 |
|  | BMI | 0.48[-0.36, 1.32] | 0.265 | 0.27[-0.69, 1.23] | 0.584 |
|  | Sex(Male) | -1.75[-3.07, -0.42] | 0.010 | -1.65[-3.53, 0.23] | 0.085 |
| **Right Ventricular Ejection Fraction (RVEF)** | hypertension | -0.10[-2.09, 1.89] | 0.920 | -0.46[-2.81, 1.88] | 0.697 |
|  | diabetes | 0.47[-4.92, 5.85] | 0.865 | -2.40[-6.01, 1.22] | 0.002 |
|  | weight | -0.14[-0.46, 0.19] | 0.411 | 0.19[-0.16, 0.54] | 0.280 |
|  | height | 0.04[-0.25, 0.33] | 0.774 | -0.21[-0.54, 0.13] | 0.230 |
|  | age | 0.08[0.03 0.13] | 0.002 | 0.05[-0.02, 0.11] | 0.174 |
|  | BMI | 0.27[-0.66, 1.19] | 0.572 | -0.49[-1.47, 0.48] | 0.321 |
|  | Sex(Male) | -2.02[-3.48, -0.57] | 0.007 | -3.39[-5.32, -1.47] | 0.001 |
| **Left Ventricular End-Diastolic Volume (LVEDV)** | hypertension | -0.03[-10.21, 10.14] | 0.995 | -3.82[-14.36, 6.73] | 0.476 |
|  | diabetes | 2.46[-25.09, 30.01] | 0.861 | 4.21[-12.04, 20.46] | 0.610 |
|  | weight | 0.08[-1.59, 1.75] | 0.926 | 0.38[-1.18, 1.95] | 0.632 |
|  | height | 2.20[0.71, 3.70] | 0.004 | 1.63[0.11, 3.15] | 0.035 |
|  | age | -0.25[-0.50, -0.00] | 0.049 | -0.53[-0.83, -0.22] | 0.001 |
|  | BMI | 1.53[-3.20, 6.27] | 0.525 | 0.47[-3.93, 4.87] | 0.832 |
|  | Sex(Male) | 14.99[7.54, 22.44] | 0.000 | 8.42[-0.22, 17.06] | 0.056 |
| **Right Ventricular End-Diastolic Volume (RVEDV)** | hypertension | 0.94[-9.91, 11.79] | 0.865 | 4.29[-8.82, 17.40] | 0.519 |
|  | diabetes | -1.03[-30.41, 28.34] | 0.945 | -2.79[-23.00, 17.42] | 0.786 |
|  | weight | -0.14[-1.92, 1.63] | 0.873 | -0.82[-2.77, 1.12] | 0.406 |
|  | height | 2.39[0.80, 3.99] | 0.003 | 2.69[0.80, 4.57] | 0.005 |
|  | age | -0.38[-0.65, -0.12] | 0.005 | -0.67[-1.05, -0.29] | 0.001 |
|  | BMI | 2.38[-2.67, 7.43] | 0.355 | 3.57[-1.90, 9.04] | 0.200 |
|  | Sex(Male) | 19.84[11.89, 27.78] | 0.000 | 20.67[9.92, 31.42] | 0.000 |
| **Left Ventricular Global Longitudinal Strain (LVGLS)** | hypertension | 1.22[0.26, 2.17] | 0.013 | 1.77[0.72, 2.82] | 0.001 |
|  | diabetes | 0.59[-1.99, 3.18] | 0.653 | 1.71[0.09, 3.33] | 0.039 |
|  | weight | 0.20[0.04, 0.35] | 0.014 | -0.12[-0.27, 0.04] | 0.148 |
|  | height | -0.16[-0.30, -0.02] | 0.026 | 0.02[-0.13, 0.17] | 0.793 |
|  | age | 0.01[-0.02, 0.03] | 0.657 | -0.03[-0.06, -0.00] | 0.042 |
|  | BMI | -0.49[-0.94, -0.05] | 0.030 | 0.20[-0.33, 0.72] | 0.458 |
|  | Sex(Male) | 1.27[0.56, 1.98] | 0.000 | 0.07[-0.99, 1.13] | 0.905 |

| **Supplementary Table 12: Summary of Cardiac Parameters in Individuals with PASC vs. Controls: Narrative Interpretation of Meta-Analysis and IPD Findings** | | | | | | |
| --- | --- | --- | --- | --- | --- | --- |
| **Cardiac Parameter** | **No. of Studies** | **Mean Difference (95% CI)** | **p-value** | **Heterogeneity (I²)** | **IPD Consistency** | **Narrative Interpretation** |
| LVEF (%) | 13 | −1.47 (−2.85 to −0.09) | <0.01 | High | Yes | Statistically significant but modest reduction in LVEF observed in the PASC group; both groups had preserved EF. |
| LVEDV (mL) | 5 | −4.87 (−8.62 to −1.12) | <0.01 | High | Yes | Lower LVEDV values were associated with the PASC group across studies. |
| LV GLS (%) | 10 | 1.17 (0.56 to 1.77) | <0.001 | High | Yes | PASC was associated with consistently lower LV GLS values, indicating differences in myocardial deformation. |
| TAPSE (mm) | 4 | −0.90 (−1.81 to 0.01) | 0.66 | Low | No | A borderline statistically significant association with lower TAPSE values in the PASC group was observed. |
| E/e′ Ratio | 5 | 0.00 (−0.42 to 0.42) | 0.21 | Moderate | Not Available | No statistically significant association between E/e′ ratio and PASC. |
| RVEF (%) | 3 | Not significant | 0.13 | Moderate | Not Available | No consistent association between RVEF and PASC across studies. |
| sPAP (mmHg) | 3 | Not significant | 0.61 | Low | Not Available | Pulmonary pressures were not significantly different between groups; no strong association identified. |
| Heart Rate (bpm) | 4 | +4.23 | <0.01 | High | Not Available | A small but statistically significant association with higher heart rate was observed in the PASC group. |
| Systolic Blood Pressure (mmHg) | 3 | −1.2 | 022 | Moderate | Not Available | No statistically significant association between systolic blood pressure and PASC. |
| Diastolic Blood Pressure (mmHg) | 3 | −1.54 | 0.11 | Moderate | Not Available | No statistically significant association between diastolic blood pressure and PASC. |
| LVEF: Left Ventricular Ejection Fraction (%) \| LVEDV: Left Ventricular End-Diastolic Volume (mL) \| LV GLS: Left Ventricular Global Longitudinal Strain (%) \| TAPSE: Tricuspid Annular Plane Systolic Excursion (mm) \| LVMI: Left Ventricular Mass Index (mg/m²) \| E/e′ Ratio: Ratio of Early Mitral Inflow Velocity to Mitral Annular Early Diastolic Velocity \| RVEF: Right Ventricular Ejection Fraction (%) \| sPAP: Systolic Pulmonary Artery Pressure (mmHg) \| Heart Rate: Beats Per Minute (bpm) \| Blood Pressure: Millimeters of Mercury (mmHg) | | | | | | |

| **Supplementary Table 13: Post Hoc Sensitivity Analysis of IPD LVEF Results With and Without Inclusion of the Australian Cohort (Marwick et al., 2024)** | | | |
| --- | --- | --- | --- |
| **Analysis Scenario** | **PASC Mean LVEF (95% CI)** | **Control Mean LVEF (95% CI)** | **Mean Difference (PASC − Control) (95% CI)** |
| **Full IPD Sample (n = 956)** | 61.36 (60.70–62.02) | 59.70 (59.39–60.20) | +1.66 (0.33 - 2.83) |
| **Excluding Marwick et al. (n = 576)** | 61.13 (60.58–61.68) | 63.03 (61.01–65.05) | −1.90 (−3.99 to 0.19) |
| **Note.** This post hoc sensitivity analysis was conducted to explore the observed discrepancy between the individual patient data (IPD) meta-analysis and the study-level meta-analysis. The Australian cohort (Marwick et al., 2024) contributed disproportionately to the pooled IPD results and reported higher LVEF in individuals with PASC compared to controls. Exclusion of this cohort reversed the direction of the effect and brought the IPD result into alignment with the findings of the study-level meta-analysis. **Caution:** As a post hoc analysis, these results are sensitive to the exclusion of a single large study and should be interpreted with caution. | | | |

| **Supplementary Table 14: Cardiovascular Impact of Long COVID: Comparison of LVGLS and LVEF in Individuals with and without Diabetes** | | | | | | |
| --- | --- | --- | --- | --- | --- | --- |
| **Group** | **GLS (%)**  **Mean (95% CI)** | **Δ (95% CI)** | **P-value** | **LVEF (%)**  **Mean (95% CI)** | **Δ (95% CI)** | **p-value** |
| **Control (No Diabetes)** | 19.4 (19.0–19.7) | **Reference** | **-** | 61.9 (61.1–62.7) | **Reference** | **-** |
| **Control (Diabetes)** | 18.7 (18.3–19.0) | -0.72(-1.2- -0.18) | 0.009 | 60.3 (59.1–61.5) | -.151(-2.98- -0.16) | 0.030 |
| **Long COVID (Diabetes)** | 15.9 (14.2–17.5) | -2.78(-4.47 - -1.08) | 0.002 | 58.9(56.82–60.9) | -1.4(-3.7-0.94) | 0.232 |
| **Long COVID (No Diabetes)** | 15.0 (14.8–15.2) | -4.3(-4.8- -3.92) | 0.002 | 59.5 (59.1–59.9) | -2.41(-3.32- -1.51) | 0.000 |
| **CI:** Confidence Interval**\| LVEF:** Left Ventricular Ejection Fraction \| **LVGLS:** Left Ventricular Global Longitudinal Strain \| **T2DM:** Type 2 Diabetes  *Mean differences and p-values were calculated for LVEF and LVGLS, across different groups, using Control (No Diabetes) as the reference group.  *Long COVID (T2DM=1) vs Control (T2DM=1); Long COVID (T2DM=0) vs Control (T2DM=0); Control (T2DM=1) vs Control (T2DM=0) | | | | | | |

| **Supplementary Table 15: Cardiovascular Impact of Long COVID: Comparison of LVGLS and LVEF in Individuals with and without hypertension** | | | | | | |
| --- | --- | --- | --- | --- | --- | --- |
| **Group** | **GLS (%)**  **Mean (95% CI)** | **Δ (95% CI)** | **P-value** | **LVEF (%)**  **Mean (95% CI)** | **Δ (95% CI)** | **p-value** |
| **Control (No HTN)** | 18.4 (17.8–19.0) | **Reference** | **-** | 61.4 (60.2–62.6) | **Reference** | **-** |
| **Control (HTN)** | 19.4 (19.1–19.7) | 0.96(0.33-1.6) | 0.003 | 61.4 (60.5–62.2) | -0.01(-1.4- 1.4) | 0.984 |
| **Long COVID (HTN)** | 15.1 (14.2–16.0) | -4.3(-5.3 - -3.3) | 0.000 | 59.7 (58.6–61) | -1.6(-3.0-0.1) | 0.031 |
| **Long COVID (No HTN)** | 15.1 (14.8–15.3) | -3.34(-3.9- -2.7) | 0.000 | 59.4 (58.9–59.8) | -1.9(-3.2- -0.7) | 0.003 |
| **CI:** Confidence Interval**\| LVEF:** Left Ventricular Ejection Fraction \| **LVGLS:** Left Ventricular Global Longitudinal Strain \| **HTN:** Hypertension  *Mean differences and p-values were calculated for LVEF and LVGLS across different groups, using Control (No HTN) as the reference group.  *****Long COVID (HTN =1) vs Control (HTN =1) ; Long COVID (HTN =0) vs Control (HTN =0); Control (HTN =1) vs Control (HTN=0) | | | | | | |

| **Supplementary Table 16: Effect Modification Analysis of LVEF and GLS** | | | |
| --- | --- | --- | --- |
| **Outcome** | **Variable** | **Coefficient (β) with 95% CI** | **p-value** |
| LVEF | Long COVID | -2.69 (-4.03, -0.91) | 0.000 |
|  | T2DM | -1.51 (-2.68, -0.34) | 0.012 |
|  | HTN | -0.10 (-1.01, 0.82) | 0.829 |
|  | Constant | 64.68 (61.64, 67.72) | 0.000 |
| GLS | Long COVID | -4.480 (-5.335, -3.626) | 0.000 |
|  | T2DM | -2.942 (-4.847, -1.038) | 0.003 |
|  | HTN | 0.651 (-1.208, 2.511) | 0.492 |
|  | Long COVID × T2DM | 1.88 (0.55, 3.22) | 0.006 |
|  | Constant | 24.009 (22.344, 25.674) | 0.000 |

| **Supplementary Table 17: Summary of egger test parameters across cardiac outcomes** | | | | | | | | | |
| --- | --- | --- | --- | --- | --- | --- | --- | --- | --- |
| **Source** | **SS** | **df** | **MS** | **Number of obs** | **F(1, 8)** | **Prob > F** | **R-squared** | **Adj R-squared** | **Root MSE** |
| **Left Ventricular Ejection Fraction** | | | | | | |  |  |  |
| Model | 0.095028448 | 1 | 0.095028448 | 10 | 0.03 | 0.8753 | 0.0033 | -0.1213 | 1.9027 |
| Residual | 28.9621704 | 8 | 3.6202713 |  |  |  |  |  |  |
| Total | 29.0571989 | 9 | 3.22857765 |  |  |  |  |  |  |
| **Left Ventricular End-Diastolic Volume** | | | | | | |  |  |  |
| Model | 0.710007008 | 1 | 0.710007008 | 5 | 0.07 | 0.8067 | 0.0232 | -0.3024 | 3.1554 |
| Residual | 29.8699941 | 3 | 9.95666471 |  |  |  |  |  |  |
| Total | 30.5800011 | 4 | 7.64500028 |  |  |  |  |  |  |
| **Global Longitudinal Strain for LV Function** | | | | | | |  |  |  |
| Model | 0.467193472 | 1 | 0.467193472 | 5 | 0.87 | 0.4203 | 0.2243 | -0.0342 | 0.73379 |
| Residual | 1.61532669 | 3 | 0.538442231 |  |  |  |  |  |  |
| Total | 2.08252017 | 4 | 0.520630042 |  |  |  |  |  |  |
| **Tricuspid Annular Plane Systolic Excursion(TAPSE)** | | | | | | |  |  |  |
| Model | 0.173712298 | 1 | 0.173712298 | 4 | 0.33 | 0.6238 | 0.1415 | -0.2877 | 0.72587 |
| Residual | 1.05378761 | 2 | 0.526893806 |  |  |  |  |  |  |
| Total | 1.22749991 | 3 | 0.409166637 |  |  |  |  |  |  |
| **systolic pulmonary arterial pressure** | | | | | | | | | |
| Model | 1.16919508 | 1 | 1.16919508 | 3 | 1.02 | 0.4969 | 0.5048 | 0.0096 | 1.0709 |
| Residual | 1.14687158 | 1 | 1.14687158 |  |  |  |  |  |  |
| Total | 2.31606666 | 2 | 1.15803333 |  |  |  |  |  |  |

| **Supplementary Table 18: Overall quality of evidence based on GRADE approach** | | |
| --- | --- | --- |
| **Domain** | **Quality rating** | **Comment** |
| Risk of bias | High | Most information is taken from studies (included studies) at low risk of bias |
| Consistency of results | Moderate | There was considerable heterogeneity among studies. However, the study explored the heterogeneity through sub-group analysis and meta-regressions |
| Directness of evidence | Moderate | Most of the included studies examined the direct impact of PASC on cardiac parameters. However, variations in how long COVID was measured and the diversity within the control groups introduced some indirectness in the evidence |
| Precision of results | Moderate | Despite small sample sizes, the studies had narrow confidence intervals, leading to a moderate precision rating. |
| Publication bias | High | Using funnel plots, Egger’s test, and the trim-and-fill technique, we found no evidence of publication bias, leading us to conclude that the quality of evidence regarding publication bias is high in confidence. |

# **Section 3: Supplementary Figures**

Supplementary Figure 1: Forest Plot of Mean Differences in E/e' Ratio (Early Diastolic Filling Velocity to Mitral Annular Velocity)

Supplementary Figure 2: Forest Plot of Mean Differences in Right Ventricular Ejection Fraction (RVEF)

Supplementary Figure 3: Forest Plot of Mean Differences in systolic pulmonary arterial pressure (sPAP [mmHg])

Supplementary Figure 4: Forest Plot of Mean Differences in Heart rate (beats per minute)

Supplementary Figure 5: Forest Plot of Mean Differences in Systolic blood pressure [mmHg]

Supplementary Figure 6: Forest Plot of Mean Differences in Diastolic blood pressure [mmHg]

Supplementary Figure 7: Heat map of case-control and cohort study quality score using the Newcastle-Ottawa Scale(NOS)

Supplementary Figure 8: Heat map of cross-sectional study quality score using the Joanna Briggs Institute (JBI)

Supplementary Figure 9: Funnel Plot Assessing Publication Bias in Studies Reporting on Left Ventricular Ejection Fraction (LVEF)

Supplementary Figure 10: Funnel Plot Assessing Publication Bias in Studies Reporting on Left Ventricular End-Diastolic Volume

Supplementary Figure 11: Funnel Plot Assessing Publication Bias in Studies Reporting on Global Longitudinal Strain for LV Function

Supplementary Figure 12: Funnel Plot Assessing Publication Bias in Studies Reporting on TAPSE

Supplementary Figure 13: Funnel Plot Assessing Publication Bias in Studies Reporting on sPAP

Supplementary Figure 14: Assessment of Publication Bias in Left Ventricular Ejection Fraction (LVEF) Studies via Trim-and-Fill

Supplementary Figure 15: Assessment of Publication Bias in Left Ventricular End-Diastolic Volume Studies via Trim-and-Fill

Supplementary Figure 16: Assessment of Publication Bias in Global Longitudinal Strain for LV Function Studies via Trim-and-Fill

Supplementary Figure 17: Sensitivity analysis of Left Ventricular Ejection Fraction (LVEF)

Supplementary Figure 18: Sensitivity analysis of Left Ventricular End-Diastolic Volume (LVEDV)

Supplementary Figure 19: Sensitivity analysis of Global Longitudinal Strain for LV Function

**
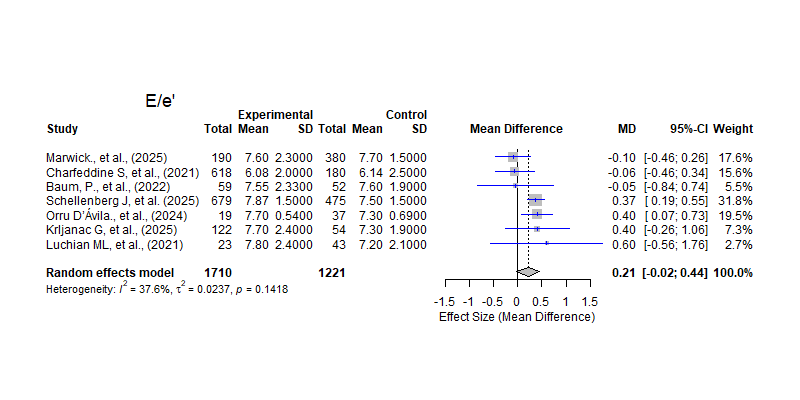
**

**Supplementary Figure 1: Forest Plot of Mean Differences in E/e' Ratio (Early Diastolic Filling Velocity to Mitral Annular Velocity)**

**
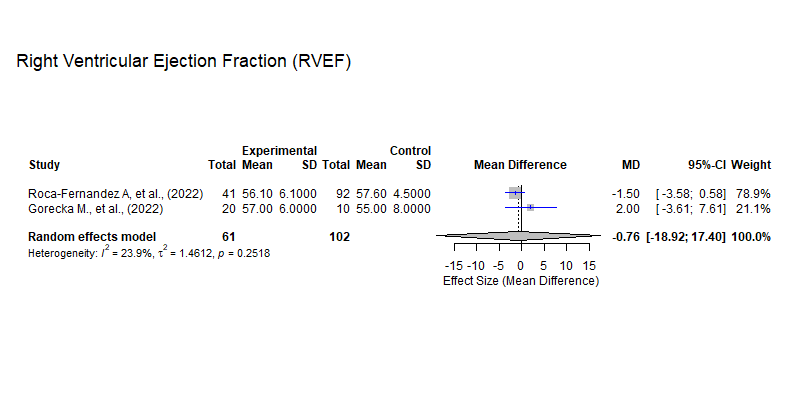
**

**Supplementary Figure 2: Forest Plot of Mean Differences in Right Ventricular Ejection Fraction (RVEF)**

**
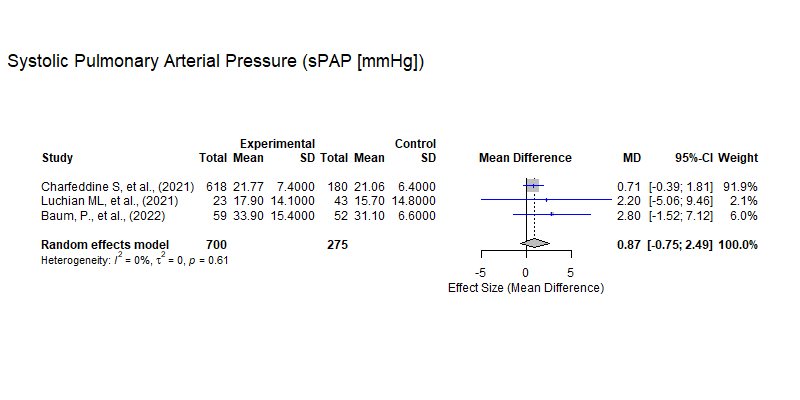
**

**Supplementary Figure 3: Forest Plot of Mean Differences in systolic pulmonary arterial pressure (sPAP [mmHg])**

**
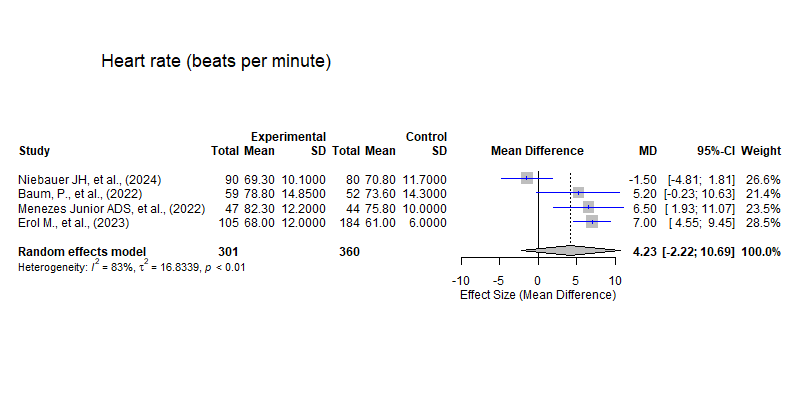
**

**Supplementary Figure 4: Forest Plot of Mean Differences in Heart rate (beats per minute)**

**
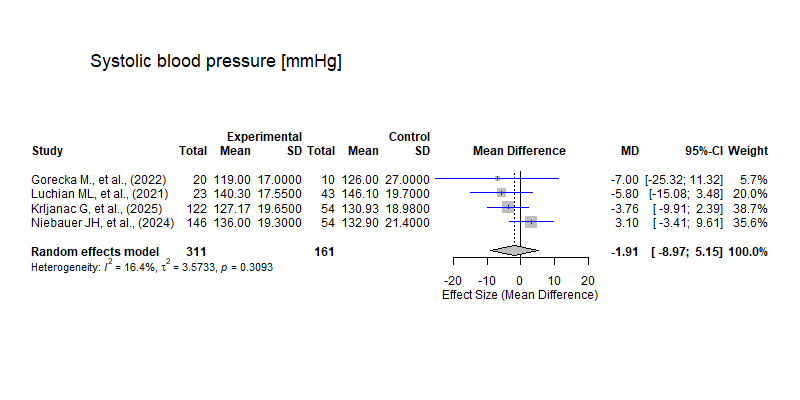
**

**Supplementary Figure 5: Forest Plot of Mean Differences in Systolic blood pressure [mmHg]**

**
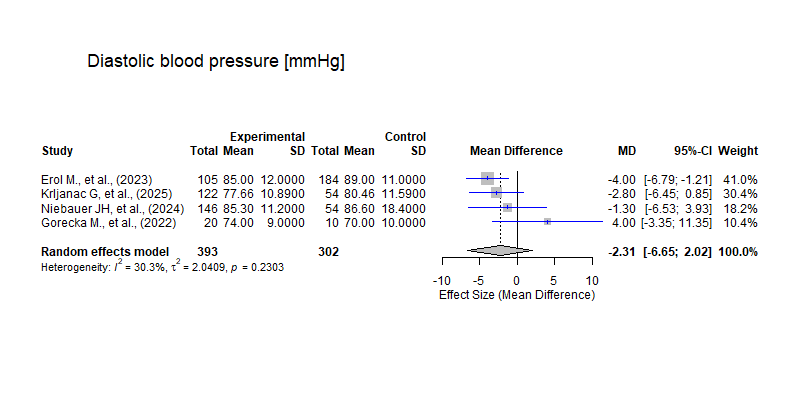
**

**Supplementary Figure 6: Forest Plot of Mean Differences in Diastolic blood pressure [mmHg]**

**
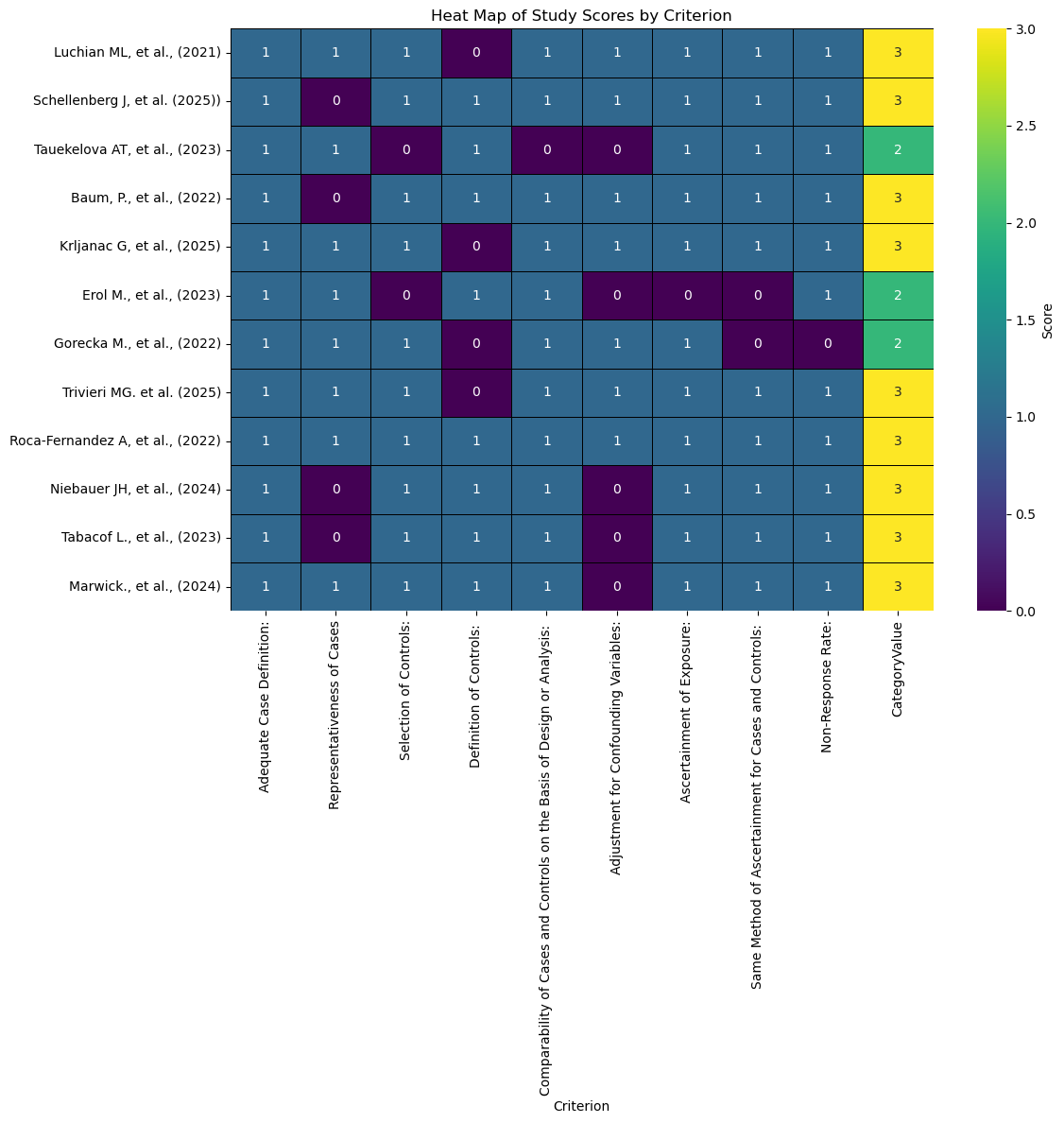
**

**Supplementary Figure 7: Heat map of case-control and cohort study quality score using the Newcastle-Ottawa Scale (NOS)**

**
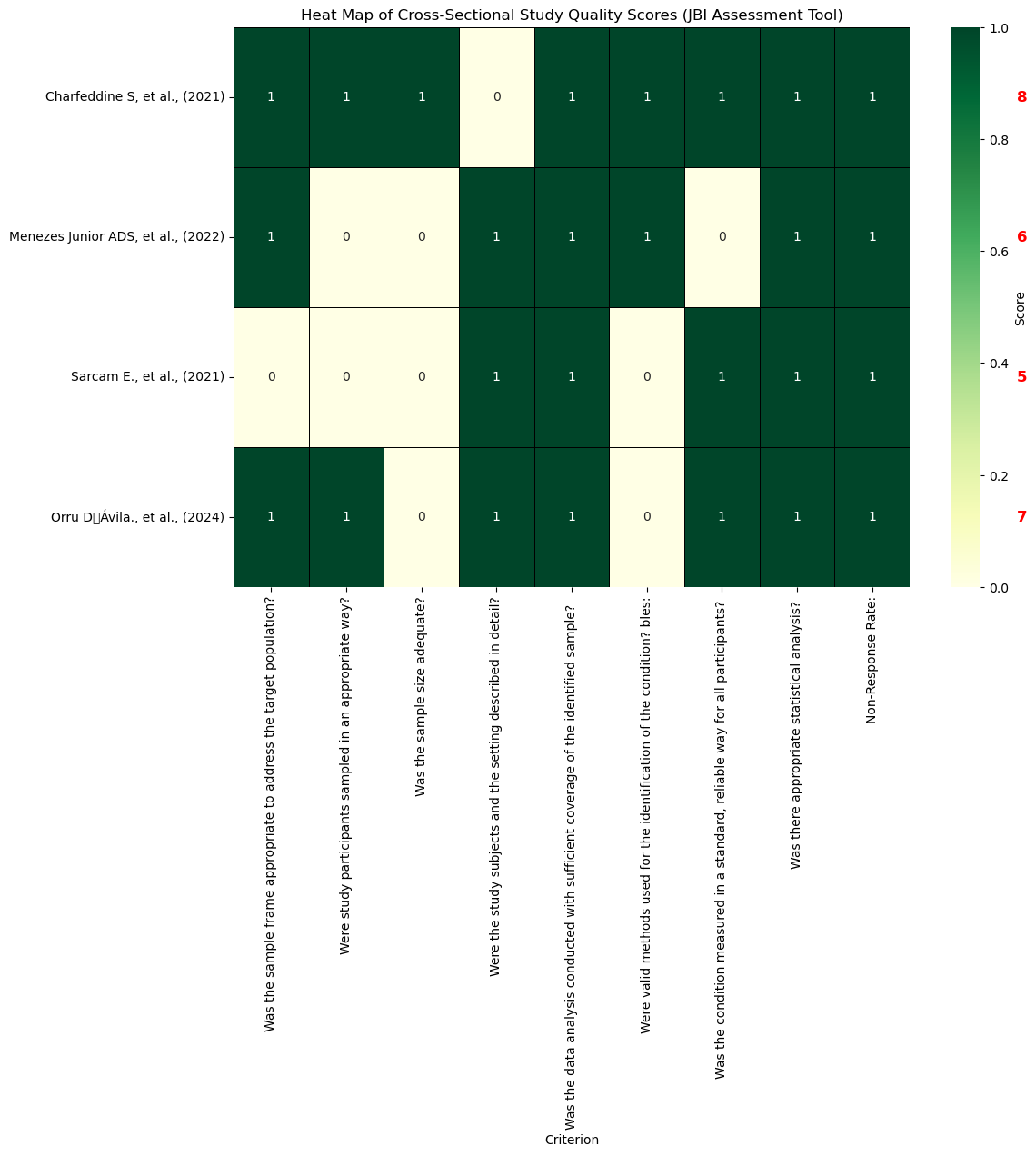
**

**Supplementary Figure 8: Heat map of cross-sectional study quality score using the Joanna Briggs Institute (JBI)**

**
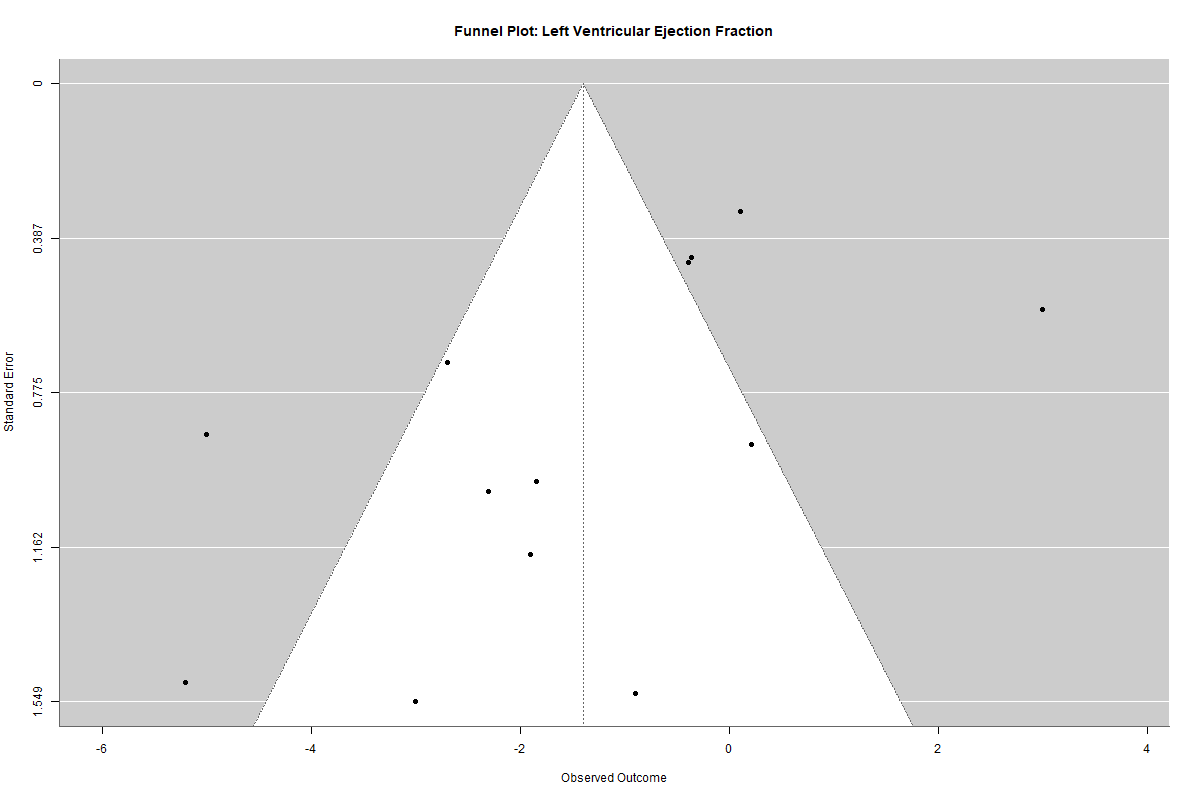
**

**Supplementary Figure 9: Funnel Plot Assessing Publication Bias in Studies Reporting on Left Ventricular Ejection Fraction (LVEF)**

**
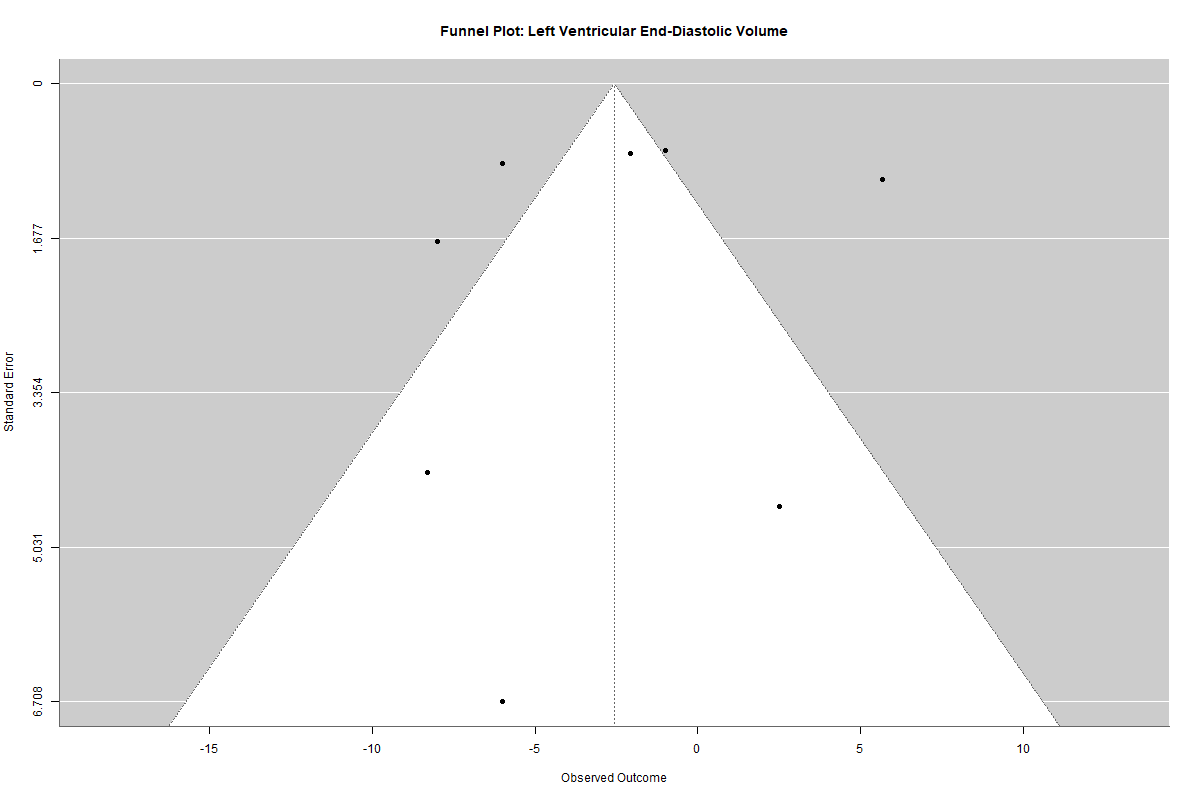
**

**Supplementary Figure 10: Funnel Plot Assessing Publication Bias in Studies Reporting on Left Ventricular End-Diastolic Volume**

**
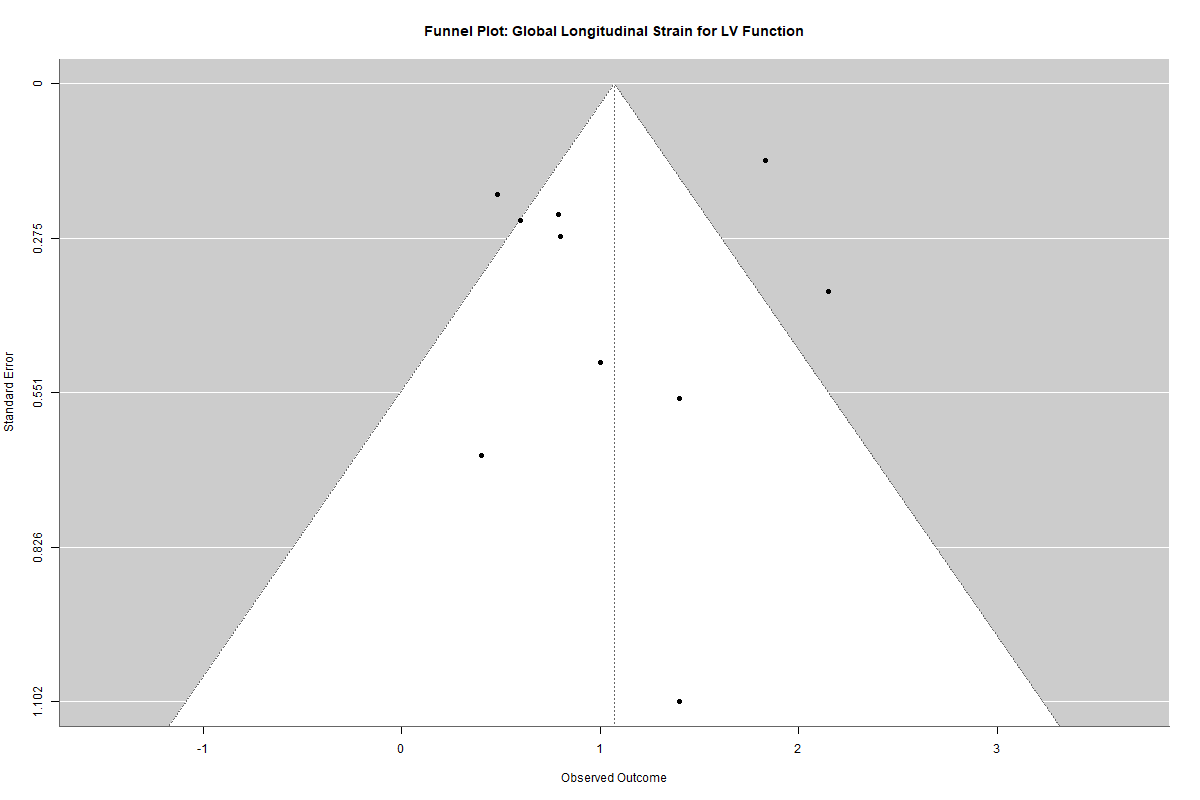
**

**Supplementary Figure 11: Funnel Plot Assessing Publication Bias in Studies Reporting on Global Longitudinal Strain for LV Function**

**
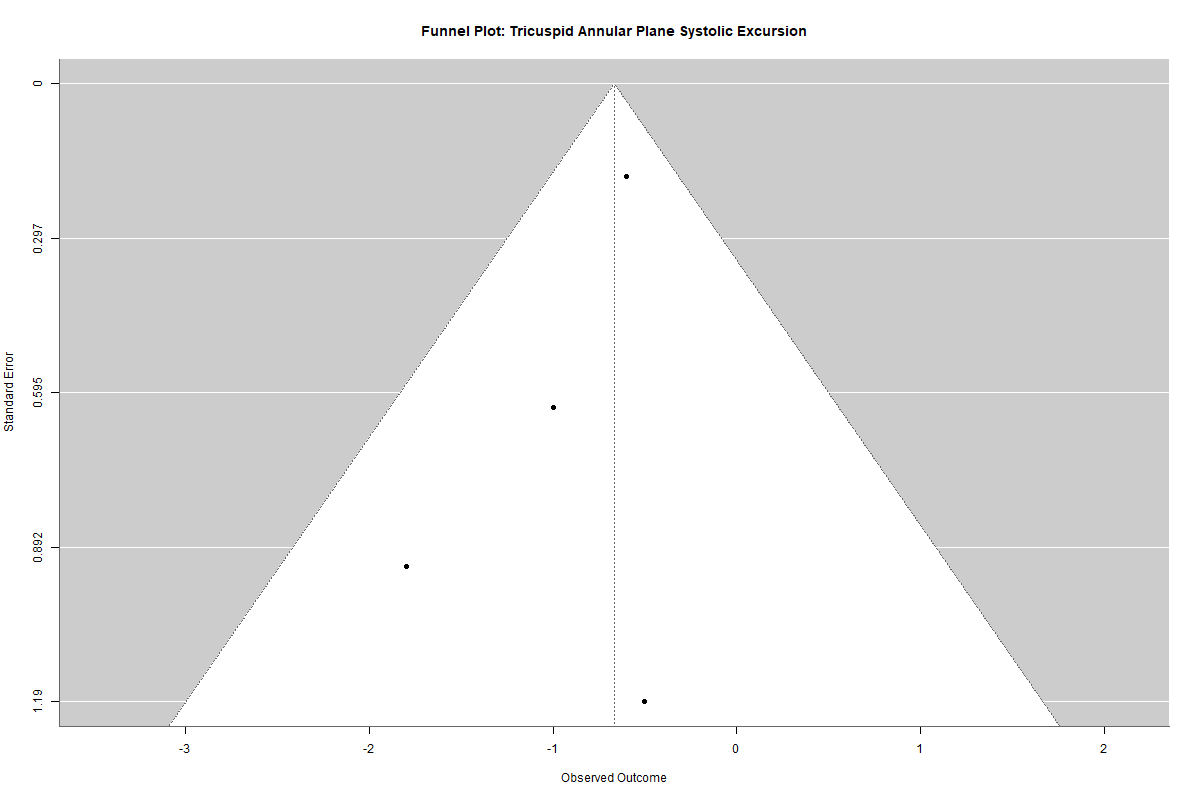
**

**Supplementary Figure 12: Funnel Plot Assessing Publication Bias in Studies Reporting on TAPSE**

**
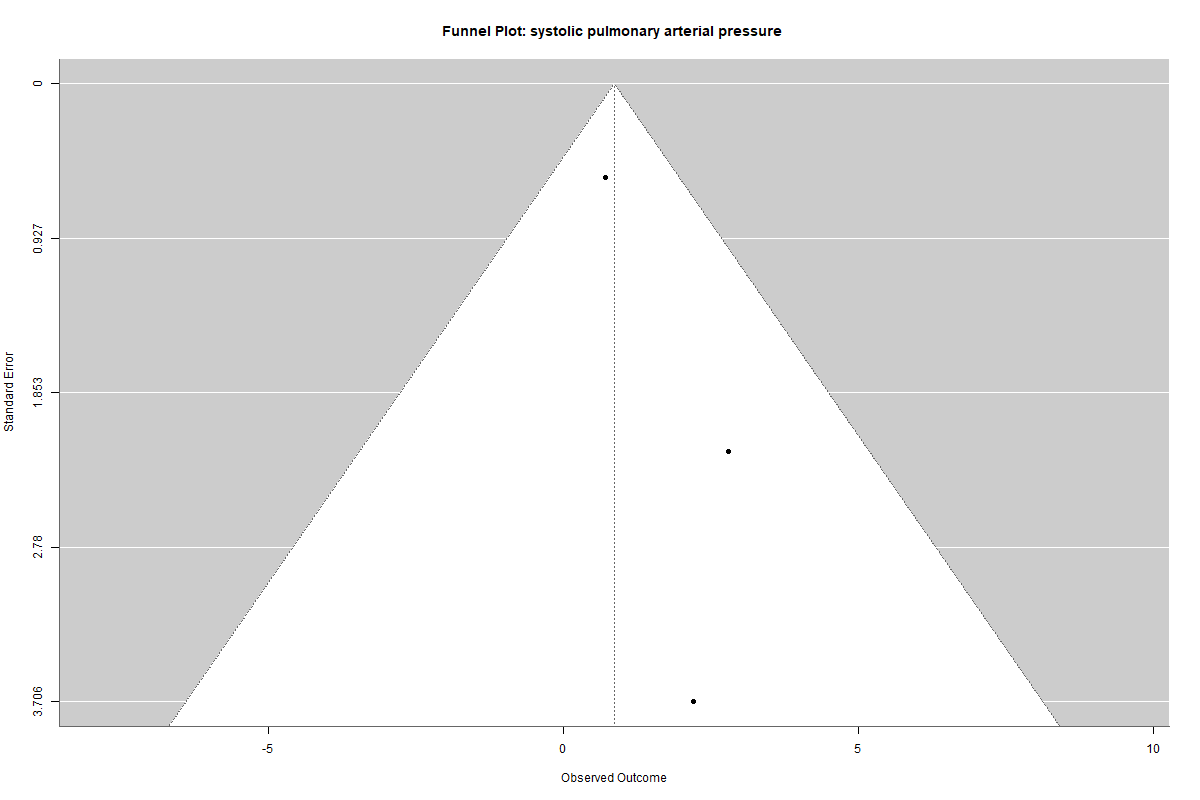
**

**Supplementary Figure 13: Funnel Plot Assessing Publication Bias in Studies Reporting on sPAP**

**
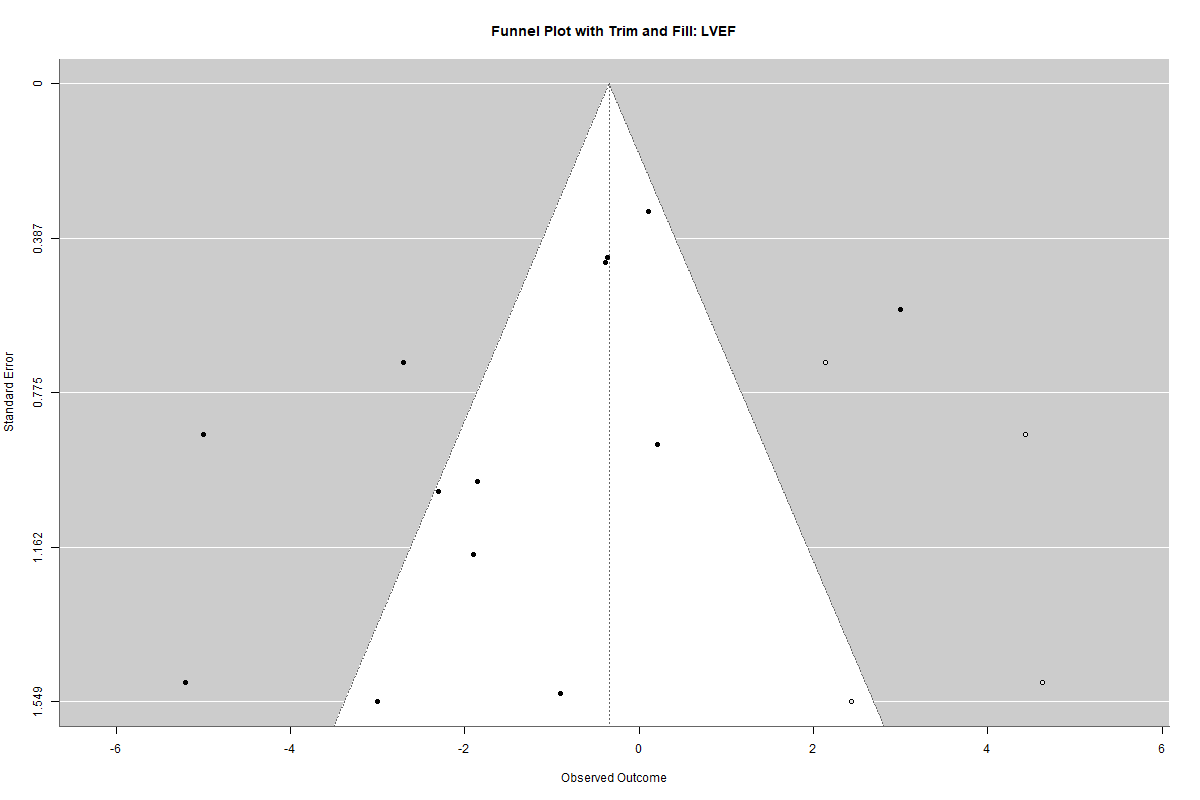
**

**Supplementary Figure 14: Assessment of Publication Bias in Left Ventricular Ejection Fraction (LVEF) Studies via Trim-and-Fill**

**
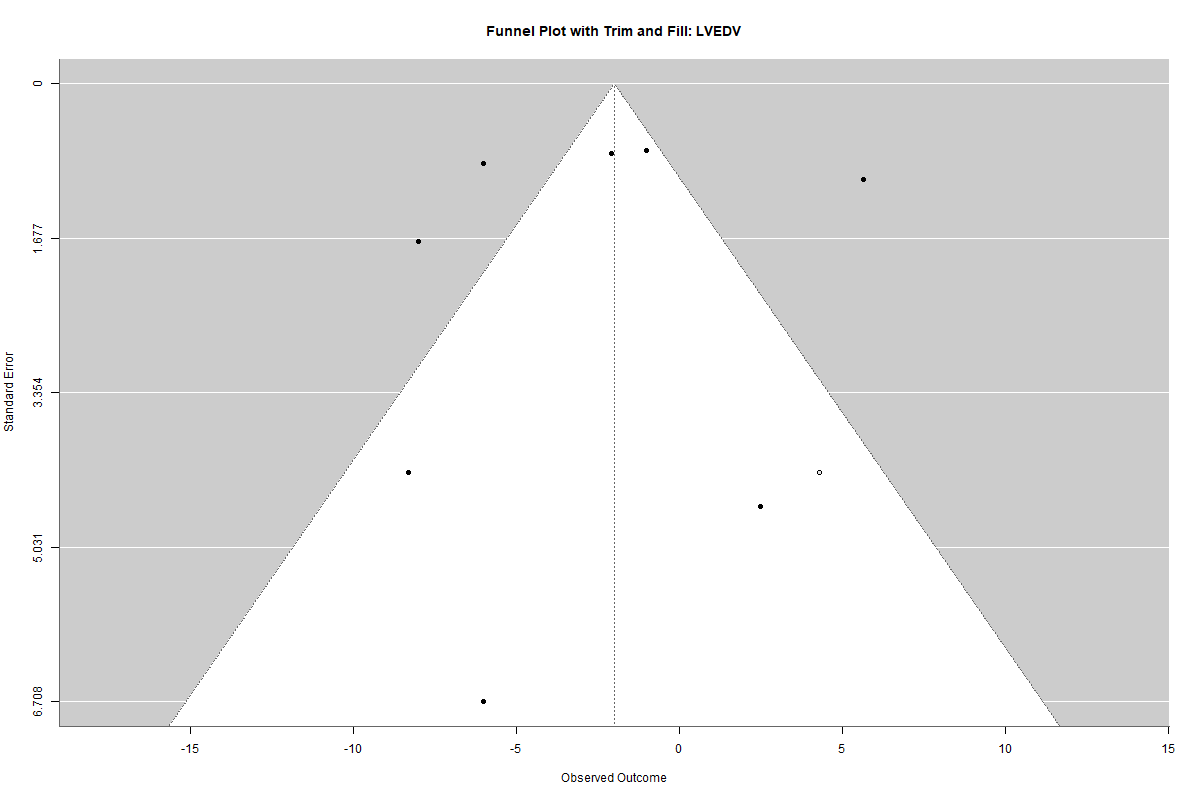
**

**Supplementary Figure 15: Assessment of Publication Bias in Left Ventricular End-Diastolic Volume Studies via Trim-and-Fill**

**
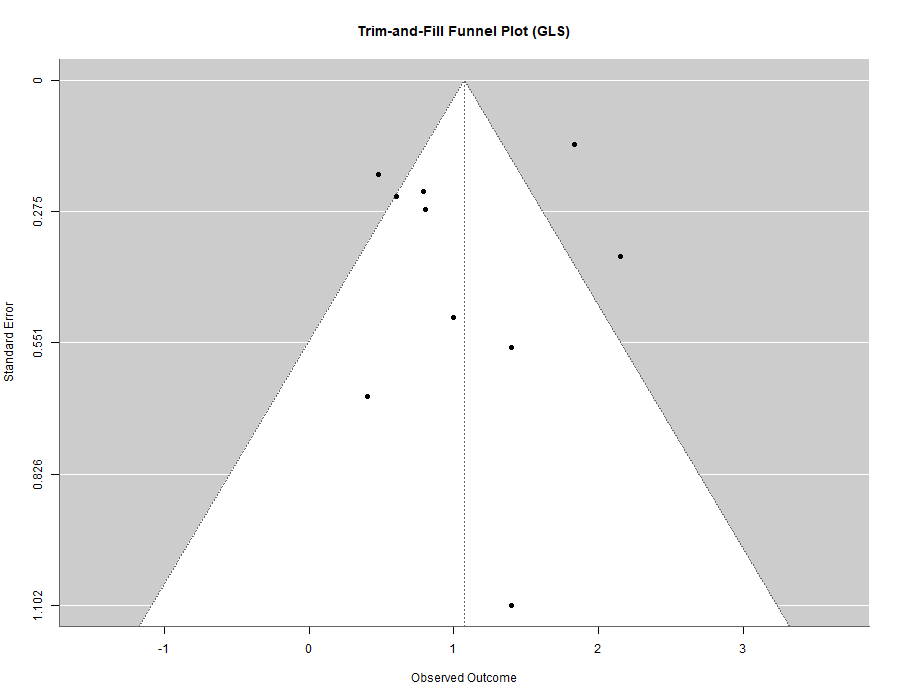
**

**Supplementary Figure 16: Assessment of Publication Bias in Global Longitudinal Strain for LV Function Studies via Trim-and-Fill**

**
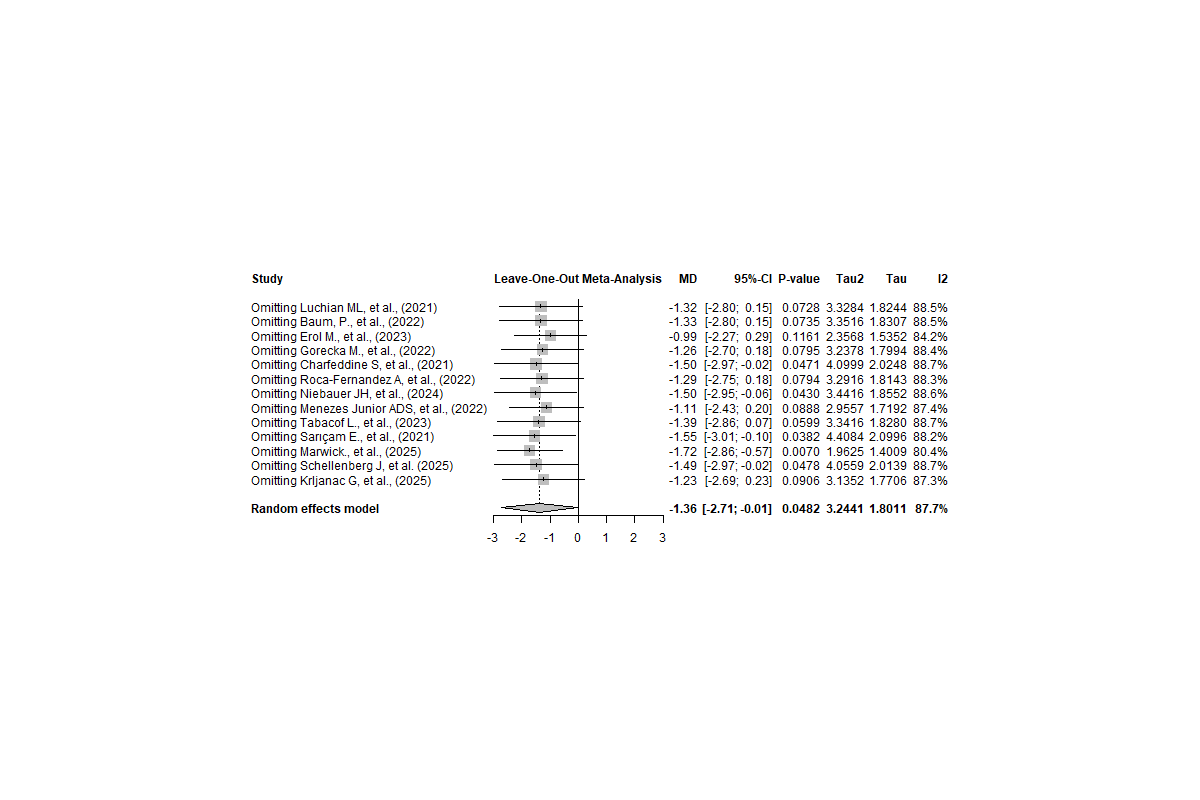
 Supplementary Figure 17: Sensitivity analysis of Left Ventricular Ejection Fraction (LVEF)**

**
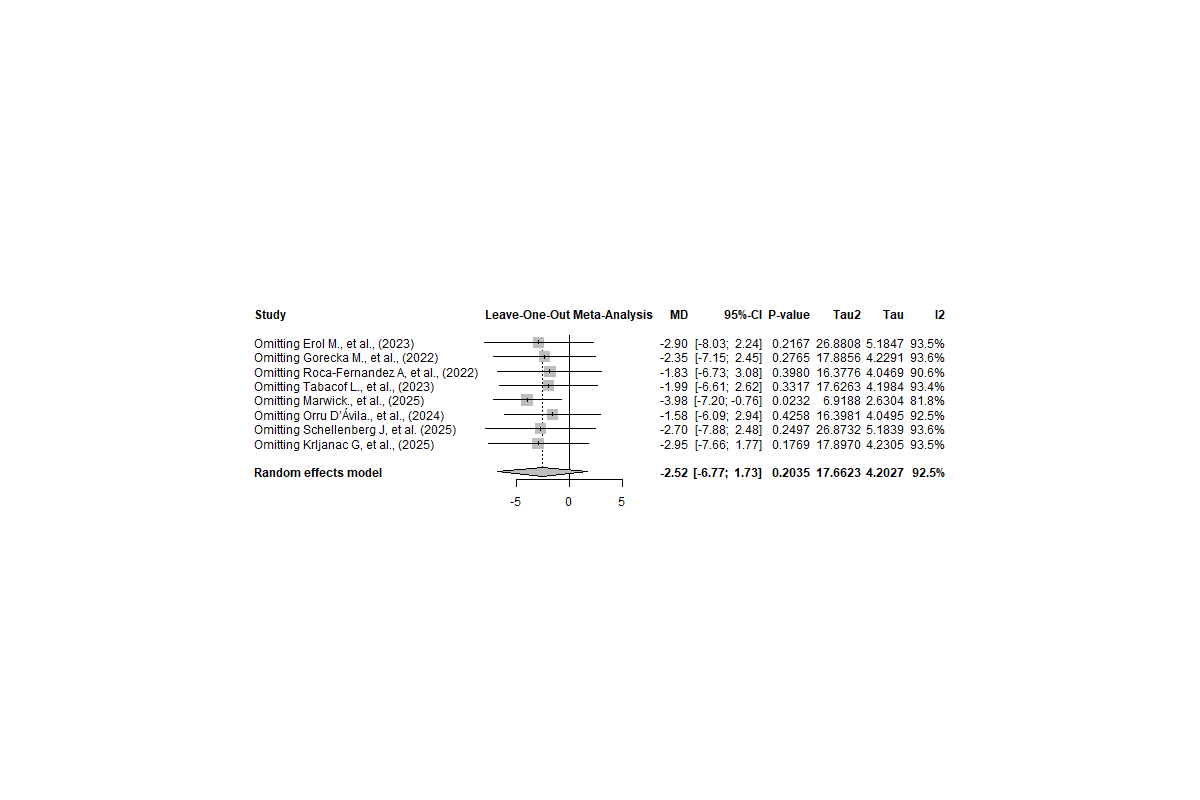
Supplementary Figure 18: Sensitivity analysis of Left Ventricular End-Diastolic Volume (LVEDV)**

**
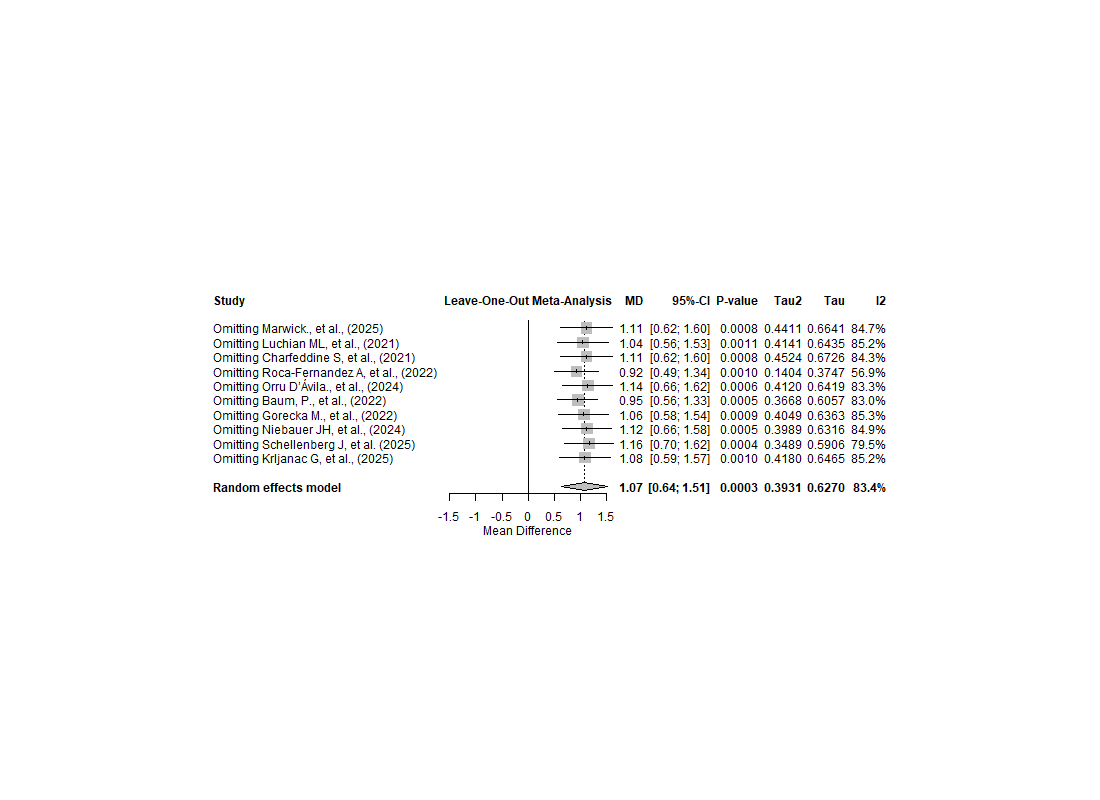
Supplementary Figure 19: Sensitivity analysis of Global Longitudinal Strain for LV Function**

# **Section 4: Cardiac imaging methodology**

**Section 4 Table**. Summary of Imaging Modalities, Parameters Assessed, and Protocols Across Included Studies

This table provides detailed descriptions of the imaging techniques (e.g., echocardiography, cardiac MRI, CT) and derived cardiac parameters used in the included studies, addressing methodological heterogeneity.

| **Study (First Author, Year)** | **Imaging Modality** | **Cardiac Parameters Reported** | **Imaging Equipment / Protocol** |
| --- | --- | --- | --- |
| **Luchian ML, 2021** | 2D TTE with speckle tracking and myocardial work analysis | LVEF, LA, FAC, TAPSE, PAP, GLS, GCW, GWW, GWE, GWI, RV | GE Vivid E9; EchoPAC v20.3 |
| **Schellenberg J, et al. (2025)** | TTE with speckle-tracking echocardiography (STE) | LV GLS, RV GLS, RV FWS, LV EF (A4C/A2C), LV EDVi (A4C/A2C), LV ESVi (A4C/A2C), LA size, RA size, E/A ratio, E/E' lateral and medial ratios, RV basal and midventricular diameters, RV length, TAPSE | 2D apical 2- and 4-chamber views |
| **Gorecka M, 2022** | CMR (cine bSSFP, T1/T2 mapping, perfusion mapping, LGE), ³¹P-CMRS | LVEF, LV/RV volumes, GLS, MBF (stress/rest), native/post-contrast T1, T2, LGE | 3T MRI scanner |
| **Tauekelova AT, 2023** | Chest CT, 2D echocardiography, resting & Holter ECG, 24h BP monitor | Echo-derived LVEF (not detailed), ECG, Holter ECG, 24h BP, 6MWT | Siemens Somatom Definition AS CT |
| **Baum P, 2022** | TTE with 2D speckle tracking | LVEF, LVEDD, LVEDV, LVESV, LAD, LAVI, RVEDD, RAA, TAPSE, sPAP, GLS | GE Vivid E95; ASE/EACVI guidelines |
| **Krljanac G, et al., (2025** | TTE with 2D speckle-tracking echocardiography (STE), CMR with LGE and T1/T2 mapping | LVEF, LS (endo, mid, epi), CS (endo, mid, epi), CS PSS, LVEDV/I, LVESV/I, E/A, e', E/e', DT, RVSP, RV parameters (RVEF, FAC, GLS), LA parameters (LAV/I, strains), CMR: LV/RV volumes and EF, LVM, T1/T2 native/post-contrast, ECV, LGE | ECG-512A (400 Hz) |
| **Erol M, 2023** | Stress–rest SPECT MPI | LVEF, wall motion, summed stress score, perfusion, coronary stenosis | Tc‑99m MIBI, Siemens dual-head SPECT |
| **Trivieri MG. et al. (2025)** | 18F-FDG PET/MRI, DECT (dual-energy CT) | Myocardial 18F-FDG uptake, LGE (nonischemic pattern), pericardial 18F-FDG uptake and LGE, periannular 18F-FDG uptake and LGE, vascular 18F-FDG uptake (aorta/pulmonary artery), pulmonary infiltrates (DECT), abnormal perfusion (DECT) | Not specified |
| **Charfeddine S, 2021** | TTE, speckle tracking, PORH | EQI, LV systolic/diastolic function, LVGLS | E4-diagnose system |
| **Roca-Fernandez A, 2022** | Multiparametric cardiac MRI (as part of multi-organ MRI) | LVEF, RVEF, LV/RV EDV, GLS, T1 mapping (≥3 segments) | Siemens 1.5T/3T |
| **Niebauer JH, 2024** | TTE, Cardiac MRI, biomarkers | LVEF (cine MRI), NT-proBNP, troponin T, 6MWT distance | Medis software |
| **Menezes Jr. ADS, 2022** | 24 h Holter ECG, chest CT | HRV parameters, ECG rhythm | DMS 300‑4L; chest CT for PASC |
| **Tabacof L., 2023** | TTE (data warehouse extraction) | LVEF, LVEDV, LVESV, stroke volume, LV mass index, LVIDd/s, RV and atrial size/function, valve morphology/function, PH, pericardial findings | Not specified |
| **Sarıçam E., 2021** | TTE, CMR (subset), Cardiac PET (subset) | LVEF, pericardial effusion, T1/T2 mapping, LGE, FDG uptake, biomarkers | Philips Affiniti 50G; 3T GE CMR |
| **Marwick, 2025** | 2D and Doppler TTE with speckle tracking | LV/RV GLS, RV free wall strain, E/A/e’, LVEF, LV volumes, LAVI, LAE, LVH, LVMI | ACUSON SC2000 (Siemens Healthcare) |
| **Orru D’Ávila, 2024** | TTE with 2D STE and MW analysis | LVEF (Simpson method), mitral inflow velocities, e’, TAPSE, s’, PASP, LVGLS, RV free wall strain, MW indices | Vivid S70N; EchoPAC |

**Abbreviations:** TTE: transthoracic echocardiography; STE: speckle-tracking echocardiography; MW: myocardial work; CMR/cMRI: cardiac magnetic resonance imaging; CT: computed tomography; PET: positron emission tomography; SPECT: single photon emission computed tomography; ³¹P-CMRS: phosphorus-31 cardiac MR spectroscopy; bSSFP: balanced steady-state free precession; LGE: late gadolinium enhancement; MOLLI: modified Look-Locker inversion recovery; MPI: myocardial perfusion imaging. **Cardiac structural and functional parameters** – LVEF: left ventricular ejection fraction; RVEF: right ventricular ejection fraction; LVEDV: left ventricular end-diastolic volume; LVESV: left ventricular end-systolic volume; LVMI: left ventricular mass index; LAD: left atrial diameter; LAVI: left atrial volume index; RAA: right atrial area; RV: right ventricle; RVEDD: right ventricular end-diastolic diameter; LA: left atrium; FAC: fractional area change; TAPSE: tricuspid annular plane systolic excursion; PASP/sPAP: pulmonary artery systolic pressure; LVH: left ventricular hypertrophy; LVIDd/s: LV internal diameter in diastole/systole; LVPWD: LV posterior wall diameter; SV: stroke volume; LVOT: left ventricular outflow tract. **Strain and myocardial work indices** – GLS/LVGLS: global longitudinal strain (left ventricle); RVLS: right ventricular longitudinal strain; GCW: global constructive work; GWW: global wasted work; GWE: global work efficiency; GWI: global work index.

# **Section 5: Supplementary Statistical Methodology**

Building upon the primary analyses described in the main manuscript, supplementary analyses were conducted to further elucidate the cardiovascular impact of Long COVID in specific subgroups and explore effect modification. All analyses were performed in **R (version 4.2.3)** using packages including metafor for meta-analyses and meta-regression.

1. **Group Comparisons (Supplementary Tables 9 and 10):** Group-level means and 95% confidence intervals (CIs) were calculated for LVEF and LVGLS across subgroups defined by Long COVID status, diabetes, and hypertension. The control group without comorbidities served as the reference. Between-group differences (Δ), 95% CIs, and p-values were estimated. Comparisons were stratified to examine Long COVID (T2DM = 1) vs Control (T2DM = 1), Long COVID (T2DM = 0) vs Control (T2DM = 0), and Control (T2DM = 1) vs Control (T2DM = 0), with similar comparisons conducted for hypertension.
2. **Effect Modification Analysis (Supplementary Table 11):** Multivariable linear regression models were used to examine associations between Long COVID, diabetes, and hypertension with LVEF and LVGLS. Interaction terms (e.g., Long COVID × T2DM) were included to assess potential effect modification. Regression coefficients, 95% CIs, and p-values were reported.
3. **Meta-Regression (Supplementary Table 12):** Random-effects meta-regression models were fitted to explore sources of heterogeneity in pooled mean differences of cardiac outcomes. Covariates included demographic and clinical factors such as age, sex, diabetes, hypertension, coronary heart disease, and ICU admission.
4. **Multivariable Regression by COVID Severity (Supplementary Table 13):** Separate regression models for mild and severe Long COVID participants evaluated associations between clinical predictors (age, sex, BMI, diabetes, hypertension, etc.) and cardiac function parameters including LVEF, RVEF, LVEDV, RVEDV, and LVGLS.
5. **Publication Bias Assessment (Supplementary Table 14):** Egger’s regression test was performed to detect small-study effects and publication bias across cardiac outcomes, with parameters such as R-squared and root mean squared error reported.

All statistical significance was evaluated at a two-sided alpha level of 0.05. The supplementary analyses complement the main findings by providing subgroup-specific and interaction effect insights to better understand the cardiovascular sequelae of Long COVID.
